# Intelligent Techniques for Detecting Network Attacks: Review and Research Directions

**DOI:** 10.3390/s21217070

**Published:** 2021-10-25

**Authors:** Malak Aljabri, Sumayh S. Aljameel, Rami Mustafa A. Mohammad, Sultan H. Almotiri, Samiha Mirza, Fatima M. Anis, Menna Aboulnour, Dorieh M. Alomari, Dina H. Alhamed, Hanan S. Altamimi

**Affiliations:** 1 Computer Science Department, College of Computer and Information Systems, Umm Al-Qura University, Makkah 21955, Saudi Arabia; shmotiri@uqu.edu.sa; 2 SAUDI ARAMCO Cybersecurity Chair, Department of Computer Science, College of Computer Science and Information Technology, Imam Abdulrahman Bin Faisal University, P.O. Box 1982, Dammam 31441, Saudi Arabia; 2180007084@iau.edu.sa (S.M.); 2180007105@iau.edu.sa (F.M.A.); 2180007190@iau.edu.sa (M.A.); 2180002223@iau.edu.sa (H.S.A.); 3 Department of Computer Science, College of Computer Science and Information Technology, Imam Abdulrahman Bin Faisal University, P.O. Box 1982, Dammam 31441, Saudi Arabia; saljameel@iau.edu.sa; 4 Department of Computer Information Systems, College of Computer Science and Information Technology, Imam Abdulrahman Bin Faisal University, P.O. Box 1982, Dammam 31441, Saudi Arabia; rmmohammad@iau.edu.sa; 5 SAUDI ARAMCO Cybersecurity Chair, Department of Computer Engineering, College of Computer Science and Information Technology, Imam Abdulrahman Bin Faisal University, P.O. Box 1982, Dammam 31441, Saudi Arabia; 2180007089@iau.edu.sa (D.M.A.); 2180007125@iau.edu.sa (D.H.A.)

**Keywords:** network security, network attacks, attack detection, machine learning, deep learning

## Abstract

The significant growth in the use of the Internet and the rapid development of network technologies are associated with an increased risk of network attacks. Network attacks refer to all types of unauthorized access to a network including any attempts to damage and disrupt the network, often leading to serious consequences. Network attack detection is an active area of research in the community of cybersecurity. In the literature, there are various descriptions of network attack detection systems involving various intelligent-based techniques including machine learning (ML) and deep learning (DL) models. However, although such techniques have proved useful within specific domains, no technique has proved useful in mitigating all kinds of network attacks. This is because some intelligent-based approaches lack essential capabilities that render them reliable systems that are able to confront different types of network attacks. This was the main motivation behind this research, which evaluates contemporary intelligent-based research directions to address the gap that still exists in the field. The main components of any intelligent-based system are the training datasets, the algorithms, and the evaluation metrics; these were the main benchmark criteria used to assess the intelligent-based systems included in this research article. This research provides a rich source of references for scholars seeking to determine their scope of research in this field. Furthermore, although the paper does present a set of suggestions about future inductive directions, it leaves the reader free to derive additional insights about how to develop intelligent-based systems to counter current and future network attacks.

## 1. Introduction and Background

Rapid advancements in technology have made the Internet easily accessible and it is now actively used by the majority of people for a plethora of professional and personal tasks. Various sensitive activities including communication, information exchange, and business transactions are carried out using the Internet. The Internet helps foster connection and communication, but the integrity and confidentiality of these connections and information exchanges can be violated and compromised by attackers who seek to damage and disrupt network connections and network security. The number of attacks targeting networks are increasing over time, leading to the need to analyze and understand them and develop more robust security protection tools. Every organization, industry, and government requires network security solutions to protect them from the ever growing threat of cyber-attacks. The need for more effective and stable network security systems to protect business and client data is rising as there is no network immune to network attacks.

Several techniques have been proposed over the years to handle and classify network traffic attacks. One is the port-based technique, which includes identifying port numbers among the ones registered by the Internet Assign Number Authority (IANA) [1]. However, due to the growing number of applications, the number of unpredictable ports has increased and this technique has proven to be ineffective. Furthermore, this technique does not cover account applications that do not register their ports with the IANA or applications that use dynamic port numbers. Another technique that has been proposed is the payload-based technique, also known as deep packet inspection (DPI), where the network packet contents are observed and matched with an existing set of signatures stored in the database [1]. This method provides more accurate results than the port-based technique, but does not work on network applications using encrypted data. Furthermore, this technique has been proven to be complex, involving high computational costs and a high processing load [1]. Behavioral classification techniques analyze the entire network traffic received at the host in order to identify the type of application [2]. The network traffic patterns can be analyzed graphically as well as by examining heuristic information, for example, transport layer protocols and the number of distinct ports contacted. Although behavioral techniques yield good results as they are able to detect unknown threats, they are resource-intensive and are prone to false positives. Another technique, called the rationale-based technique or the statistical technique [2] examines the statistical characteristics of traffic flow, namely, the number of packets and the maximum, mean, and minimum of the packet size. These statistical characteristics are used to identify different applications since these measurements are unique for every application. However, there is a growing need to incorporate this approach with techniques that could improve the accuracy and speed up the process of classifying the statistical patterns. The correlation-based classification [2] accumulates packets into flows; that is, it collects data packets with the same source and destination IP, port, and protocol. These are classified according to the correlation between network flows. Multiple flows are usually accumulated further into a Bag of Flow (BoF). Although this technique has proven to perform better than statistical techniques as it overcomes the problem of feature redundancy, it has a high computational overhead for feature matching. Therefore, the need to create techniques that could overcome the rising challenges persist.

At the onset of the 21st century, the concepts of intelligent techniques, namely machine learning (ML) and deep learning (DL) became widespread. Researchers widely acknowledged that these techniques could greatly increase the calculation potential since they focus on using statistical methods and data to make computers think the way humans think. Hence, these intelligent techniques started being used by computer scientists in network security as they addressed the limitations of the non-intelligent techniques. In the field of network security, ML or DL algorithms can be trained with network data to recognize traffic type as normal or malicious and thus protect the network from intruders. Furthermore, the algorithms can be trained to identify the attack type if the network traffic is malicious and trigger appropriate action to prevent the attack. By analyzing past cyber-attacks, the model can be taught to prepare individual defensive reactions. These applications of intelligent methods in network security, which is the focal point of this research paper, can be useful in big businesses, organizations, law enforcement agencies, and banks that store sensitive information as well as in personal networks.

In the past, most of the developed network attack detection techniques actively depended on a set of pre-defined signature-based attacks. This was a major setback since the database of the attacks needed to be constantly updated as the attackers found new ways to exploit network security. However, with the evolution of intelligent-based techniques such as ML and DL, the predictive accuracy of identifying and classifying network attacks has been greatly improved. Therefore, using intelligent-based techniques in network security is a thriving field for research that needs to be explored.

Although several review articles exploring how intelligent-based systems have been applied to detect network attacks have been published in the last few years, none have been found that are as comprehensive as this article. This article covers almost one hundred research articles produced from 2010 to 2021 on a range of network attacks. It will provide clear insights into the race between developing intelligent systems to counter network attacks and how these attacks have evolved to circumvent intelligent systems, thus highlighting gaps in the research and indicating potential future research areas. This research also applied a different taxonomy that, to the best of our knowledge, has not been used in any previous research. It sets up several criteria against which the articles being reviewed could be assessed and compared including:

(i) What is/are the classification algorithms implemented?
(ii) What is/are the datasets employed for developing the intelligent systems?
(iii) Furthermore, this research article compared the results obtained using different evaluation metrics.

It then discusses the answers to the following main questions:

(i) Which algorithm(s) was/were commonly implemented and in which kind of attacks?
(ii) Which dataset(s) is/are considered more reliable based on the results obtained?

The resulting comparisons and discussions will help future researchers to identify the directions to take in their research, that is, to either improve the intelligent-based algorithms or consider other algorithms, to identify the features that should be added or removed when building the training dataset, and to indicate the evaluation metrics that should be adopted to evaluate the created intelligent systems.

The outcomes of this paper provide valuable directions for further research and applications in the field of applying effective and efficient intelligent techniques in network analytics.

This article is organized into four sections. The first section provides an introduction and background to the research area. A brief overview of network attacks is presented in Section 2. Section 3 discusses intelligent network attack mitigation techniques where all the reviewed research papers, the network attacks they address using ML and DL techniques, and their findings are presented. Finally, the last section provides a discussion of the findings and the ideas presented in the papers reviewed and sets out promising research directions.

## 2. Network Attacks

For decades, networking technologies have been used to improve data transfer and circulation. Their continuous improvements have facilitated a wide range of new services.

The Internet of Things (IoT) is a powerful tool for improving communication by connecting different devices to the Internet and collecting data. The information gathered assists firms in the analysis and forecasting of consumer behavior to enhance the quality of their products. Nowadays, ML and DL are being used to construct network systems that can conduct advanced analytics and automation. This technology is transforming the users’ networking experiences by simulating human intellect and gathered data with built-in algorithms [3].

The emerging cloud computing technologies have brought about remarkable evolutions in network technology where different applications, services, and computing and storage resources are offered on demand to a large number of users via the Internet, thus offering tremendous advantages including flexibility, minimal administrative efforts, cost effective resource utilization, high accessibility, efficiency, and reliability [4].

A new global wireless standard is the 5th generation (5G) mobile network, which represents a logical network type that connects essentially anything including machines, objects, and gadgets. Not only does 5G offer faster speeds and a greater number of linked devices, it also enables network slicing. Network slicing is the process of dividing several virtual networks operating on the same network infrastructure to create subnetworks that meet the demands of various applications. From entertainment and gaming to school and community safety, the 5G network technology has the ability to develop anything. 5G has the potential to provide higher download rates, real-time replies, and improved connection over time, allowing companies and consumers to explore new innovations [5].

Such an exponential growth in network technologies has offered many advantages and has greatly improved communications. However, each emerging network technology presents new security challenges and triggers the need for the development of detection tools and countermeasures to meet the new demands. The following subsections briefly discuss the main types of network attacks.

### 2.1. Types of Network Attacks

A network attack is an approach to hurt, reveal, change, destroy, steal, or obtain illegal access to a network system resource. The attack could come from inside (internal attack) or from outside (external attack). Table 1 lists and describes a number of different types of network attacks that disrupt communication, classifying them as either active or passive attacks, bitcoin attacks, account attacks, or a security breach [6].

### 2.2. Network Attack Detection and Prevention Techniques

Security and defense systems are designed to identify, defend, and recover from network assaults. Confidentiality, availability, and integrity are the three primary aims of network security systems. Network intrusion detection and prevention techniques can be classified based on the approach used to detect network threats, prevent them, or a combination of both. These techniques are developed as software, hardware, or a combination of both. They can be classified into two classes: intrusion detection systems (IDS), and intrusion prevention systems (IPS) [6,7].

- Intrusion Detection System (IDS): Referred to also as network-based IDS (NIDS). This system intensely monitors malicious network activities and notifies officials if an attack is detected with no prevention abilities. Signature-based and anomaly-based detection are the two most prevalent approaches used by IDS to identify threats. Signature-based procedures are applied to detect only known threats, relying on a database containing a list of pre-existing characteristics of known attacks (attacks signatures) to identify suspicious events. The database needs to be continuously updated to include emerging attacks. On the other hand, anomaly-based procedures attempt to differentiate malicious traffic from real traffic based on a change in the network traffic; thus, they can detect unknown threats. Inconsistencies such as high-size traffic, network latency, traffic from uncommon ports, and abnormal system performance, all represent changes in the normal behaviors of the system and can indicate the presence of network attacks.
- Intrusion Prevention System (IPS): Known also as intrusion detection and prevention systems (IDPS). It scans the network continuously for the presence of illegal or rogue control points that are detected on the basis of changes in behavior. The system automatically takes countermeasures to tackle the threats and defend the system. The primary objective of an IDPS is to keep malicious or undesired packets and attacks from causing any harm. IDPS is more effective than IDS as it not only detects threats, but is able to take action against them. There are two types of IDPS: network-based intrusion detection and prevention systems (NIDPS) that analyze the network protocol to identify any suspicious activities and host-based intrusion detection and prevention systems (HIDPS) that are used to monitor host activities for any suspicious events within the host.

To identify attacks effectively and efficiently, a variety of detection approaches are constantly being developed based on intelligent techniques including ML and DL, which have recently gained immense popularity in the network security field.

## 3. Intelligent Network Attack Mitigation Techniques

In this section, research studies that used intelligent models to detect different cyber-attack types are reviewed and their findings summarized. Several ML algorithms have been used in these studies including classification, regression, and clustering techniques such as logistic regression (LR), decision trees (DT), etc. Some used random forest (RF), an ensemble of DT, in order to visually represent the sequences of the decision-making process in the form of a tree. Support vector machine (SVM) was widely used in classification due to its ability to distinctly classify the data points by building a hyperplane in an n-dimensional space, where n represents the number of features. Another ML classifier that has been widely used is naïve Bayes (NB), a supervised learning model that uses Bayes’ theorem of probability. Finally, some researchers have used the K-nearest neighbor (KNN) for classification and K-means clustering, an unsupervised approach. Further details about these algorithms can be found in [8].

DL is a subset of ML, which is a subset of artificial intelligence (AI). A number of DL techniques have been used to build the detection models in some studies, primarily the artificial neural network (ANN), which is an information-processing system that consists of several layers that work best with non-linear dependence and recurrent neural network (RNN), a type of ANN that contains memory function to maintain previous content. Another commonly used DL technique is the convolutional neural network (CNN), which is also a type of ANN that mimics human vision. Furthermore, deep neural network (DNN), a supervised learning type of ANN that finds correct mathematical manipulation to turn input into output, has been used by some authors. Long-short term memory (LSTM), a type of RNN designed to model temporal sequences more accurately, and multi-layer perceptron (MLP), a type of ANN that consists of many layers in directed graphs, have also been widely used. Finally, the gated recurrent unit (GRU), which, though a variant of LSTM and is considered to be more efficient than LSTM as it uses comparatively less memory and executes faster, has also been used. More information about the mentioned algorithms can be found in [9].

### 3.1. Problem Domains of the Reviewed Articles

The papers were classified according to the cyber-attack type on which they focused. The different attack types mentioned in this section are insider threat, DDoS attacks, zero-day attacks, phishing attacks, malware attacks, and botnet attacks. We then reviewed articles that did not target specific attacks, but aimed to identify attacks at IoT networks, classify the malicious traffic to different attacks, and identify attacks at the DNS level. Finally, we also mention papers targeting the detection of intrusions in the network.

#### 3.1.1. Insider Threat

Cybersecurity measures have tended to focus on threats outside an organization rather than threats inside that can cause harmful effects. Therefore, researchers have started to look at different techniques to identify insider threats. Tuor et al. [10] built a model using principal component analysis (PCA) for feature selection, and unsupervised DL namely, DNN, RNN, SVM, isolation forest, DNN-Ident, DNN-Diagnosis LSTM-Ident, LSTM-Diagnosis, among others, that use system logs to detect anomalous activities in the network. The dataset used was synthetic CERT insider threat v6.2 [11], which was taken from the event log lines of a network of a simulated organization’s computer. The researchers targeted two prediction approaches: the “next time step” and the “same time step”. The results of the experiments showed that the “same time step” approach resulted in higher performance, and that the isolation forest model was the strongest model. To evaluate the proposed model, recall was used and DNN-Diagnosis, LSTM-Diagnosis, and the isolation forest model all obtained 100% recall. In future work, the researchers may apply the proposed model to a wider range of streaming tasks and explore different granularities of time.

Similarly, LSTM and CNN techniques were used by Yuan et al. [12] to build a model to detect insider threats. They applied the model on the CERT insider threat v4.2 dataset [13], which contained 32 M log lines among which 7323 were anomalous activities. The advantage of this version of the CERT dataset was that it contained more samples of insider threats than other versions. The train–test split was 70–30%. The researchers first used LSTM to extract the user behavior, abstracted temporal features, and produced the feature vectors. After that, the researchers transformed the feature vectors into fixed-size matrices. Finally, CNN was used to classify the feature matrices into anomaly or normal. The proposed model resulted in an area under the curve (AUC) of 94.49%.

Hu et al. [14] used DL methods to build a user authentication model based on characteristics of mouse behaviors that could be used to monitor and detect insider authentications. They used an open-source dataset called the Balabit Mouse Dynamics Challenge dataset [15], and CNN algorithm. CNN showed high performance in user authentication based on mouse features with a false acceptance rate (FAR) of 2.94% and a false rejection rate (FRR) of 2.28%.

#### 3.1.2. DDoS Attacks

One of the most harmful threats in network security is distributed denial of service (DDoS) attacks that attempt to disrupt the availability of services. Since DDoS is easy to launch but not easy to detect, as in most cases the attack traffic is very similar to legitimate traffic, some researchers have focused solely on detecting them using different ML approaches.

Yuan et al. [16] proposed DeepDefense, which is a DL-based DDoS attack detection approach that can study network traffic sequence patterns and trace the network attack activities. They used the UNB ISCX intrusion detection evaluation 2012 (ISCX2012) dataset [17], and the RNN algorithm to build the model. From ISCX2012, the team extracted 20 network traffic fields to generate a 3-D feature map using a sliding time window. Data14 and data15 were extracted from ISCX2012, which contained 9.6 M packets and 34.9 M packets, respectively. The total number of training samples in data14 and data15 were 15,176 and 233,450, respectively. The experiment results showed that the DL models reduced the error rate by 39.69% compared to ML methods in a small dataset. For large datasets, the reduction in the error rate ranged from 7.517% to 2.103%. For future work, they suggested increasing the diversity of DDoS vectors and system settings to test the DeepDefense model as well as compare DeepDefense against other ML algorithms.

A study proposing a model for analyzing and detecting DDoS attacks on the network-level and service levels of the bitcoin ecosystem was carried out by Baek et al. [18]. The dataset consisted of real DDoS attacks [19] and contained the service affected, date of the attack, category of service, number of posts, etc. From the bitcoin block data, the researchers extracted statistical data such as maximum, minimum, summation, and standard variation. The researchers used PCA to perform feature extraction. MLP was used to detect DDoS while the training set, validation set, and testing set were divided according to the ratio 6:2:2. The results showed that the accuracy of DDoS attack detection was about 50% and the accuracy for classifying normal block data was about 70% while setting the unit of epoch to 100. In future work, the researchers wish to find out how to extract the features that impact the characteristics of the blocks made when a DDoS attack occurs.

Sabeel et al. [20] used DNN and LSTM for binary prediction of unknown DoS and DDoS attacks. To train the models, they used the CICIDS2017 dataset (size 283 MB) [17]. For testing, a new dataset called ANTS2019 (size 330 MB), which mimics real-life attacks, was generated in a simulated environment to measure performance. In feature engineering, 78 features were used for the training set and 77 for testing (the ‘Fwd Header length’ feature was dropped). The train–test split was 75–25%. When the model was trained using CICIDS2017 and part of ANTS2019, the highest evaluation accuracy of 99.68% for DNN was obtained. When the researchers demonstrated the retraining of the models on a dataset with new unknown attacks, the true positive rate (TRP) obtained was 99.8% and 99.9% for DNN and LSTM, respectively. To maintain performance, it was concluded that the models should be updated with new attacks at regular intervals.

An intrusion detection system (IDS) used against DDoS attacks called DDoSNet was built by Elsayed et al. [21], which was a combination of autoencoder (AE) with RNN. In their paper, the researchers evaluated their classifier using the newly released CICDDoS2019 dataset [22], which contained 80 flow features. For feature engineering, PCA was applied, and the input features were 77. The total number of samples for training, validation, and testing sets were 161,523, 46,150, and 23,000, respectively. When the model was evaluated, the results indicated an accuracy of 99%, outperforming all compared ML methods—SVM, DT, NB, RF, Booster, and LR. In future work, the researchers intend to test the performance of their model in different datasets and extend the work to multiclass classification, since, in this research, a binary classification framework was applied.

A model that exploited the characteristics of CNN to classify the traffic flows as either benign or malicious was proposed by Doriguzzi-Corin et al. [23]. The CICIDS2018, CICIDS2017, and ISCX2012 datasets, which can be obtained through the Canadian Institute for Cybersecurity of the University of New Brunswick (UNB), were used by the researchers. They extracted 37,378 DDoS flows, and 37,378 randomly selected benign flows from ISCX2012. Then, they repeated the process for CICIDS2017 with 97,718 for benign and 97,718 for DDoS flows, and again for CICIDS2018 [17] with 360,832 for benign and 360,832 for DDoS flows. Following the pre-processing phase, each dataset was split as 90–10% train–test sets. The results showed that the accuracy for each dataset was 99.87%, 99.67%, and 98.88%, respectively. The UNB201X dataset was then constructed by combining splits from every year and the accuracy for the model with the UNB201X dataset was 99.46%. In future work, the researchers would like to optimize the pre-processing tool, rather than the detection model and also extend the dataset’s labels.

Ahuja et al. [24] used various DL algorithms to detect the DDOS attacks: CNN, RNN, LSTM, CNN-LSTM, support vector classifier-self organizing map (SVC-SOM), and stacked autoencoder-multi layer perceptron (SAE-MLP). The team used the dataset provided by leading India Project Mentor [25], which consists of 22 features. Two different optimizers were used: stochastic gradient descent (SGD) for the first 10 epochs and Adam for the next 150 epochs. For an unencrypted network, using a CNN, traffic features can be extracted automatically. Finally, they evaluated the model using the following metrics: accuracy, precision, recall, F-score, false positive rate (FPR), and false negative rate (FNR). The highest classification accuracy of 99.75% was achieved with the SAE-MLP algorithm.

A study conducted by Shi et al. [26] focused on using DL for both packet-wise and period-wise methods for traffic DDoS attack detection. They proposed a model that leveraged a DL approach for DDoS detection, which was DeepDDoS. It used spark as a big data processing framework. Additionally, for feature selection, maximal information coefficient and mutual information were used. The LSTM model was used for the training phase due to its better performance in longer sequences. The proposed work tried to filter out the abnormal flow with the least computational costs. The dataset used was CICIDS2017 (Size 283 MB). The results showed that the model achieved over 99% accuracy when receiving five packets in a continuous flow.

A model that used DL for the detection of multi-vector DDoS on a software-defined network was construed by Quamar Niyaz et al. [27]. An SAE-based DL approach was applied and the team collected network traffic from a real network (packets for normal traffic were captured from network connected to the Internet) and a private network (packets with DDoS attacks were captured from a private lab network) for the evaluation of the model. They divided the dataset files into training and testing, and then normalized them using max–min normalization. For comparison, models with soft-max and neural networks (NN) were also developed. The result showed that SAE performed better than the soft-max and NN model. The model achieved 95.65% accuracy. The researchers intend to develop a NIDS in future to detect the DDoS along with other attacks as well as the use of DL for feature extraction from raw bytes.

Pande et al. [28] aimed to build a ML model to detect DDoS attacks. To build the proposed model, a DDoS attack was performed using the ping of death technique and detected using RF. The dataset used by the researchers was the NSL-KDD [29] dataset containing a training set of 125,973 records and testing set of 22,544 instances and 41 attributes. The building time of the model was 8.71 s and the testing time was 1.28 s. The proposed model built using the random forest (RF) algorithm resulted in 99.76% accuracy. For future work, the researchers will implement the DL technique to classify the instances.

Radivilova et al.’s [30] goal was to analyze the main methods of identifying DDoS attacks through network traffic using the SNMP-MIB dataset [31]. They used RF as the classification method. The experiments began with the training and evaluation of a time series classifier. Recurrence analysis was used to extract features and the Hurst exponent was set at 10 intervals during the experiment. The main evaluation metrics were accuracy, FNR, and TPR. A numerical experiment showed that early detection is plausible when the average attack ratio represents 15–20% of the average traffic.

Likewise, Filho et al. [32] presented a smart detection system for DoS using ML. The goal was to detect both high- and low-volume DDoS attacks. The researchers used RF, perceptron, AdaBoost, DT, SGD, and LR. Since RF achieved higher precision while using 28 variables, it was used for classifying the network traffic. The evaluation of the proposed system was based on four intrusion detection benchmark datasets, namely, CICIDS2017, CICDoS2017 [33], CICIDS2018, and customized datasets. To evaluate the proposed model, recall, precision, and F-measure (F1) were used. In the CICIDS2018 and CIC-DoS2017 datasets, the proposed system achieved precision and a detection rate (DR) of more than 93% with a false alarm rate (FAR) of less than 1%. The researchers intend to include an analysis of DDoS attacks of Heartbleed and brute force attacks in their future work and to evolve methods for correlating triggered alarms.

Correspondingly, Vijayanand et al. [34] proposed a detection system of novel DoS attacks using multi-layer deep algorithms arranged in hierarchical order to detect the attacks accurately by analyzing the smart meter network traffic. The suggested technique addresses issues arising as a result of a large amount of input data and the complexity of input features. To evaluate the designed model, 9919 records from the CICIDS2017 dataset were used. The performance of the proposed system was analyzed by comparing it with simple multi-layer DL algorithms and hierarchical SVM algorithms, obtaining efficiency values of 39.78% and 99.99%, respectively.

An improved rule induction (IRI) based model was put forth by Mohammed et al. [35] for detecting DDoS attacks. UNSW-NB15 [36] dataset was used and, following the application of under-sampling without replacement and further pre-processing as well as correlation-based feature selection, the final dataset ended up with eight attributes. The suggested algorithm, called IRI for detecting DDoS attacks (IRIDOS), eliminates all insignificant items during the model creation and reduces the searching space to create the classification rules. Furthermore, the algorithm stops learning a rule after reaching a ‘rule-power’ threshold. The proposed technique was evaluated on 13 datasets from the UCI repository. IRI obtained a F1 score of 93.90% on UNSW-NB15. The model attained promising results, especially when compared to other data mining algorithms such as PRISM (divide-conquer knowledge-based approach), PART (a rule-based classification algorithm), and OneRule (OR).

An evaluation and comparison of the performance of different supervised ML algorithms on the CAIDA DoS attack dataset [37] were carried out by Robinson and Thomas [38]. Other datasets used were CAIDA Conficker, and KDD-99 [39]. The different ML algorithms included NB, RF, MLP, BayesNet, J.48, IBK, and Voting. It was observed that since the CAIDA Conficker dataset contained DDoS attacks generated from large botnets with flooding-attack vectors that were easily distinguishable with more bias, all ML algorithms, except NB, achieved an accuracy rate of more than 99% in this dataset.

Research that used the same CAIDA dataset was conducted by Barati et al. [40] who developed a hybrid ML technique to detect DDoS attacks. The CAIDA USCD 2007 was used for the attack as it contained an hour of anonymized traces from a DDoS attack on 4 August 2007. For normal traffic, the CAIDA Anonymized 2013 was used as it contained passive traces from CAIDA passive monitors in 2013. For feature selection and attack detection, genetic algorithm (GA) and ANN were used, respectively, and to select the most efficient feature wrapper method, GA was applied. The attack detection method was improved by deploying the MLP method of ANN. While building the model, the 10-fold cross-validation technique was used. The results showed that the proposed method obtained an excellent AUC of 99.91%. The researchers’ future work will include performing more experiments to detect the robustness of the model on different datasets.

Kim et al. [41] developed a model based on a convolutional neural network (CNN) for DoS attacks. They used two different datasets: the KDD-99 dataset and the CICIDS2018 dataset. They generated two types of intrusion images, RGB and grayscale. They considered the number of convolutional layers and the size of the kernel when they designed their CNN model. They performed both binary classification and multiclass classification. Moreover, the performance of the proposed model was evaluated by comparing it to the recurrent neural network (RNN) model. The best results were achieved with the KDD dataset by the CNN model that showed 99% or more accuracy in the binary and multiclass classifications. The RNN showed 99% accuracy in the binary classification. The CNN model proposed by the researchers was better able to identify specific DoS attacks with similar characteristics than the RNN model.

Finally, an approach to detect DDoS attacks using GRU was carried out by Rehman et al. [42]. The team produced a high-efficiency approach called DIDDOS to detect real world DDoS attacks using GRU, a form of RNN. Different classification models, namely GRU, RNN, NB, and SMO, were applied on the CICDDoS2019 dataset. For DDoS classification in the case of reflection attacks, the highest accuracy level of 99.69% was achieved while for the DDoS classification in the case of exploitation attacks, the highest accuracy level of 99.94% was achieved using GRU.

#### 3.1.3. Phishing Attacks

Some studies have focused on training models and testing them to detect phishing attacks. For instance, the main goal of Alam et al. [43] was to defend against phishing attacks by developing an attack detection model using RF and DT, which are ML algorithms. For ML processing, a traditional phishing attack dataset from Kaggle that contained 32 features was used. To analyze the dataset characteristics, the intended model used PCA, a type of feature selection algorithm. An accuracy level of 97% through RF was reached. With less change and variance in RF, the over-fitting obstacle was controlled. Future studies will include the prediction of phishing attacks from the registered attacks in a dataset by applying CNN and implementing the IDS.

To identify phishing website attacks, a self-structuring neural network based on ANN was developed by Mohammad et al. [44]. Phishing-related features are crucial in detecting the kind of web pages that are extremely dynamic, thus the structure of the network should be constantly improved. The proposed approach addresses this issue by automating the network structuring process and demonstrating high acceptance for noisy input, fault tolerance, and significant prediction accuracy. This was accomplished by increasing the learning rate and expanding the hidden layer with additional neurons. The goal of the developed model was to obtain generalization ability, which means that the training and testing classification accuracy should be as similar as possible. The dataset included 600 legal and 800 phishing websites, with 17 characteristics retrieved using their own tool [45,46]. The accuracy of the training, validation, and testing sets were 94.07%, 91.31%, 92.18% for 1000 epochs, respectively. The principle of the model was to use an adaptive scheme with four processes including structural simplicity, learning rate adaptation, structural design adaptation, and an early stopping approach based on validation faults.

Trial and error is one of the most popular techniques used to train a neural network, but it has a significant drawback in that it takes a very long time to set the parameters and might even require the assistance of a domain expert. Rather than trial and error, a better self-structuring neural network anti-phishing model, which makes it simpler to structure NN classifiers, was proposed by Thabtah et al. [47]. The goal of the technique was to build a large enough structure from the training dataset to develop models that can be generalized to the testing dataset. During the training phase, the algorithm dynamically modifies the structural parameters in order to generate accurate non-overfitting classifiers. With a dataset of over 11,000 websites from UCI, the neural network characteristics were updated as the classification model was being built, but they were largely dependent on the computed error rate, intended error rate, and underlying technologies. When compared to Bayesian networks and DT, the findings indicated that the dynamic neural network anti-phishing model had a higher prediction accuracy. The highest average accuracy achieved was 93.06% when information gain was used for pre-processing.

A two-layered detection framework to identify phishing web attacks by using features derived from domain and DNS packet-level data was built by Rendall et al. [48] using four ML models, namely MLP, SVM, NB, and DT. The team investigated the use of the approach where a phishing domain was classified multiple times, with additional classification being carried out only when it scored below a predefined confidence level set by the owner of the system. The model was evaluated on a dataset created by the team, and it contained 5995 phishing records and 7053 benign records. After applying the models in the two-layered architecture, the highest accuracy of 86% was achieved by MLP and DT.

Li et al. [49] built a stacking model using URL and HTML features to detect phishing web pages. They used lightweight HTML and URL features as well as HTML string embeddings to make it possible to detect phishing in real-time. The 50K-PD dataset that contained around 49,947 samples as well as the 50K-IPD dataset that contained 53,103 web page samples were made and used. The stacking model was made by combining GBDT, XGBoost, and LightGBM in multiple layers. The model achieved an accuracy of 97.30% on the 50K-PD dataset and an accuracy of 98.60% on the 50K-IPD dataset.

Phishpedia, an ensemble deep learning model described in [50], addresses major technological difficulties in phishing detection by identifying and matching brand logo variations. Three different datasets were used for this experiment. First, researchers collected the first dataset by subscribing to a service; then they collected the second one from a top-ranked Alexa list, and finally, to evaluate the detection model, they collected the third dataset from a benign dataset. As a Siamese neural network converts image to vector, which assists in estimating the correlation between two visuals, this model was chosen by the researchers for their project. A better accuracy level and less runtime cost were achieved with Phishpedia. Unlike many other approaches, phishing data are not required for training. With an accuracy of 99.2%, Phishpedia outperformed the state-of-art approaches such as LogoSENSE, EMD, and PhishZoo by a large margin. In the future, the researchers plan to expand Phishpedia by adding a system to monitor phishing online.

Supervised machine learning models were used to detect phishing attacks based on novel combination features that were extracted from the URL by Batnaru et al. [51]. The researchers used a dataset from Kaggle [52] and PhishTank [53] containing 100,000 URLs that consisted of 40,000 benign URLs from Kaggle and 60,315 phishing URLs from PhishTank for the training. They used five ML models, namely MLP, RF, SVM, NB, and DT. In terms of model selection, RF was found to be the best candidate based on F1 scores. The evaluation process was performed using an unbalanced dataset that consisted of 305,737 benign URLs and 74,436 phishing URLs to evaluate the selected model in a realistic scenario. The achieved accuracy was 99.29%. The results were compared with the performance of Google Safe Browsing (GSB), which is the default protection that is available through popular web browsers. The model outperformed the GSB. In future work, the researchers’ aim is to explore the effectiveness of their model on other datasets as well as experiment with more features. They also plan to assess the robustness of the methodology against adversarial attacks that are mostly used by malicious parties.

PhishDump, a new mobile app based on a mix of LSTM and SVM algorithms, was suggested by Rao et al. [54] to detect genuine and fake websites in mobile platforms. Because PhishDump concentrates on extracting characteristics of URLs, it offers important benefits in comparison with previous efforts including quick calculation, class independence, and resistance to unintentional malware installation. The data were gathered from three separate inputs: Alexa, OpenPhish, and PhishTank. The application’s positive aspect is that it is free of external code and databases, allowing for the identification of malicious websites in as little as 621 ms. The characteristics extracted from the LSTM model are supplied as input for URL classification to SVM using a python code. Using several datasets, this application was compared against current baseline classifiers. PhishDump surpassed all previous studies with an accuracy of 97.30%. This approach has limitations such as the chance that an intruder might circumvent the approach by implementing structural modifications to the URL, and the system could miss phishing websites with shortened URLs.

Marchal et al. [55] reviewed phishing attack problems. The researchers provided guidelines for designing and evaluating phishing webpage detection techniques. They also presented the strengths and weaknesses of various design and implementation alternatives with regard to deployability and ease of use. Moreover, they provided a list of guidelines to evaluate the proposed solutions following the selection of representative ground truth, appropriate use of the dataset, and the relevant metrics. These recommendations can also enable comparison of the accuracy of different phishing detection technologies. The researchers state that academic research in phishing detection should adopt design and evaluation methods that are relevant to real-world publication.

Similarly, Das et al. [56] also reexamined the existing research on phishing and spear phishing from the perspective of different security domains such as real-time detection, dataset quality, active attacker, and base rate fallacy. They elucidated on the challenges faced and surveyed the existing solutions to phishing and spear phishing. Their work helps guide the development of more robust solutions by examining all the existing research on phishing.

#### 3.1.4. Zero-Day Attacks

Interestingly, some researchers have focused on identifying zero-day attacks. One such study was conducted by Beaver et al. [57] who used ML methods that are able to distinguish between normal and malicious traffic. In their study, they used the adaptive boosting (AdaBoost) ensemble learner with DT in order to distinguish and classify the type of traffic on the KDD-99 dataset. The implementation that was tested in this study had four levels: (1) the top-level model that puts a cap on the FPR; (2) the first internal model that includes the AdaBoost ensemble, (3) the second internal model that implements the DT, and the lowest model that provides a judgment on whether the traffic was normal and relies on an anomaly detection algorithm. The system was able to detect 82% of the attacks that were previously missed by the signature-based sensor, detected 89% of attacks that it had not been trained to detect, and had a DR of 94% and a 1.8% false alarm rate. The future goals of the researchers are to scale the performance, which will require more parallelism in the architecture and modification of the training in order to accommodate larger datasets.

Ahmed et.al. [58] proposed a DL model that was used for identifying zero-day botnet attacks in real-time with a feed-forward backpropagation ANN technique and DNN. An important factor for obtaining high performance is a reliable dataset and hence the CTU-13 dataset [59] was obtained from the Botnet Capture Facility. There were nine input layer features and the dataset size was 10,000 randomly chosen flows. The first step was to normalize the whole data followed by the application of Adam’s optimizer in the model. The train–test split was 80–20%. The result showed that the accuracies achieved were over 99.6% after 300 epochs and that the model outperformed the NB, SVM, and backpropagation algorithms. In future work, the researchers suggest examining the efficiency of the proposed model with various other datasets.

#### 3.1.5. Malware Attacks

Barut et al. [60] aimed to compare the ML algorithms, namely SVM, RF, and MLP, to determine the most accurate and the fastest method to detect malware encrypted data. Two datasets were generated: dataset1, which was produced using Stratosphere IPS [61] extracting 20 types of malware classes (Adload, Ransom, Trickbot, etc.), and dataset2, which used CICIDS2017. In feature engineering, 200 flow features were extracted and the chi-square was used. The researchers concluded that RF was the best performing algorithm as its results showed a DR of 99.996% and a FAR of 2.97%. Generally, the results showed that the SVM, RF, and MLP models are the most accurate, with some trade-offs. For dataset1, the RF model was the best performing across all evaluation metrics except for the prediction speed, which was higher when using the SVM model. For dataset2, the SVM model was the most accurate.

Marin et al. [62] developed a model for malware traffic detection of an encrypted network using DL. The specific DL model proposed in this study was the DeepMAL, which automatically discovered the best features/data representation from raw data. The dataset used was the USTCTFC2016 [63], which comprised two sections labelled malicious or normal traffic and 10 types of malware traffic. Two types of representations were used for the raw data: packets and flows. It was concluded that using raw flows representation of the input for the DL models achieved better results. The results showed that DeepMAL detected Rbot botnet with an accuracy of 99.9%, while Neris and Virut achieved 63.5% and 54.7% each. Despite the low rates achieved, they still performed better than RF.

Park et al. [64] evaluated the recognition performance of various types of attacks including IDS, malware, and shellcode using the RF algorithm and the Kyoto 2006+ [65] dataset (total size 19.8 GB). The dataset consisted of three class types: attack, shellcode, and normal. For the first two classes, there are three attack types: IDS, malware, and shellcode. This dataset contains the traffic data collected from November 2006 to December 2015. In the data preparation step, the researchers selected one month of data (May 2014) to train the model and another month (April 2014) to test the model. In the experiment, Park et al. considered 17 features and normalized the data. The overall performance was 99% for F-Score. However, it was observed that the performance of detecting different attacks differed. They propose to further evaluate the performance of the detection of various attack types using the same dataset but varying the training conditions.

In order to classify new malware variants accurately, David et al. [66] used DL to build a model using a deep belief network (DBN) algorithm that could generate and classify a malware signature automatically. The dataset used to build the proposed model was collected by the authors and contained 1800 instances and six malware categories (Zeus, Carberp, Spy-Eye, Cidox, Andromeda, and DarkCome) with 300 variants for each category. The DBN had eight layers with the output layer containing 30 neurons. The training process was unsupervised with 1200 vectors for training and 600 vectors for testing. To denoise the autoencoders, the noise ratio was 0.2 and training epochs was 1000. The model resulted in an accuracy of 98.6% when evaluated.

Reinforcement learning continuously mimics attackers to produce new malware samples, thereby giving viable attack models for defenders, as Wu et al. [67] explained. They suggested the gym-plus model, where gym-malware is improved by adding additional activities to the action space and allowing it to modify harmful portable executable files. Additionally, it retrains the algorithm using the public EMBER [68] dataset to substantially increase the DR. In gym-plus, the DQN, SARSA, and Double DQN algorithms were used, and DQN established better policies than the other algorithms. Through retraining on the adversarial instances provided by the DQN agent, malware detection accuracy increased from 15.75% to 93.5%.

Another dataset called MTA KDD 19 [69] was explored by Letteri et al. [70], who applied dataset optimization strategies to detect malware traffic. Two dataset optimization strategies, namely dimensional reduction technique based on autoencoders (AE-optimized) as well as feature selection technique based on rank relevance weight (RRw-optimized) and sensibility enhancement on the MLP algorithm were used. In RRw, feature selection consisted of two steps: dataset tampering where 5-fold cross-validation was applied, and backward feature elimination. In the AE-optimized technique, 33 input and output neurons were made and the train–validation split was 85–15%. The training set was further split to 15% testing. The highest accuracy of 99.60% was achieved in the RRw-optimized MTA KDD 19 dataset.

#### 3.1.6. Malware Botnet Attacks

A novel scheme using supervised learning algorithms and an improved dataset to detect botnet traffic was carried out by Ramos et al. [71]. Five ML classifiers were evaluated namely, DT, RF, SVM, NB, and KNN on two datasets: CICIDS2018 and ISOT HTTP [72] Botnet (total size 420 GB). A network flow metrics analysis and feature selection was carried out on both datasets after which the ISOT dataset had 20 attributes including sources, destination port numbers, and transfer protocols among the selected features, and CICIDS2018 had 19 similar kinds of attributes. Five-fold cross-validation was applied and 80% of botnet instances were used for training and the remaining for testing. For the CICIDS2018 dataset, RF and DT achieved the highest accuracy of 99.99%. For ISOT HTTP, again, RF and DT achieved a high accuracy of 99.94% and 99.90%, respectively.

Using a similar dataset, Pektas and Akerman [73] utilized DL techniques and flow-based botnet discovery methods to identify botnet using two datasets: CTU-13 and ISOT HTTP, containing both normal and botnet data. They combined two DL algorithms namely, MLP and LSTM. In feature extraction, a flow graph was constructed where all flow data were processed to extract the features. The ISOT dataset consisted of two types of botnets, namely Waledac and Zeus, whereas CTU-13 contained seven botnet families. For the ISOT dataset, the approach achieved an F-score of 98.8%, and for CTU-13, an F-score of 99.1%.

#### 3.1.7. Detecting Attacks over IoT Networks

As the Internet of Things (IoT) has become an important aspect of our lives, concerns about its security have increased, motivating researchers to focus their efforts on identifying new techniques to detect different attacks and increase the security of IoT. One such study was conducted by Abu Al-Haija et al. [74], where they developed an intelligent detection and classification DL-based system by leveraging the power of CNN for cyber-attacks in IoT communication networks. For evaluation, the NSL-KDD, which includes all the key IoT computing attacks, was employed. This system was validated and evaluated using K-fold and confusion matrix parameters, respectively. The outcome was an efficient and intelligent deep-learning-based system that can detect the mutations of IoT cyberattacks with an accuracy level that is greater than 99.3% and 98.2% for the binary-class and the multiclass, respectively. Discussions on future work include developing new software that catches and investigates data packets that communicate through the IoT environment and updating the existing dataset for more attacks.

By utilizing unique computing resources in a regular IoT space and applying an instance of extreme learning machine (ELM), a blockchain-based efficient solution for safe and secure IoT was proposed by Khan et al. [75]. This approach analyzes the credibility of the blockchain-based smart home in terms of the fundamental security objectives of confidentiality, accessibility, and integrity. The simulation outputs were provided to show that ELM’s overheads were minor in comparison to the cybersecurity advantages it brings. The ELM architecture is made up of input layers, numerous hidden layers, and a final output layer, with hidden layers consisting of fixed neurons to boost the network’s efficiency. To minimize the error rate, the backpropagation approach is combined with a feed-forward mechanism to modify the network weights. After pre-processing the data, to remove abnormalities and lessen the risk of faults, input data from NSL-KDD was mainly split into 85% training and 15% validation. The researchers aim to investigate more datasets and architectures in the future, because the presented ELM surpassed previous ML algorithms and achieved an accuracy of 93.91%.

Ullah et al. [76] aimed to detect malware-infected files and pirated software across the IoT network using the DL approach. The dataset used was collected by Google Code Jam (GCJ) [77]. The combined DL-based approach comprised two steps. First, to detect the pirated features, the TensorFlow neural network was proposed. The unwanted details were removed using the tokenization process and extra features were mined using stemming, root words, and frequency constraints. Second, to detect the malware, a new methodology based on CNN was proposed. The raw binary files were converted to a color image to solve the detection of malware by using an image classification problem. Grayscale visualization was gained by transforming the color images, which were then used to classify malware types. The results showed that this method performed better than modern methods when it came to measuring cybersecurity threats in IoT. In future work, the researchers intend to put forward an algorithm that can detect unknown malware families.

A model that was used for the classification of attacks in IoT networks and anomaly detection was created by Tama and Rhee [78] using a DNN. The team used CIDDS-001 [79], UNSW-NB15, GPRS-WEP [80], and GPRS-WPA2 [80] datasets and compared the results. The results showed a good performance in attack detection. The average performance of DNN was validated using 10-fold cross-validation on the UNSW-NB15, CIDDS-001, GPRS-WEP, and GPRS-WPA2 datasets that resulted in 94.17%, 99.99%, 82.89%, and 94% accuracy, respectively. In future work, the researchers want to investigate a larger value of trial repetition given the unaffected performance of the different validation methods.

To mitigate IoT cybersecurity threats in a smart city, Alrashdi et al. [81] proposed an anomaly detection-IoT system using the RF model of ML. The UNSW-NB15 dataset was selected for this project, which includes 49 features and nine attack classifications to revise normal and abnormal behaviors. The resulting model could detect cyber-attacks at fog nodes in a smart city by monitoring the network traffic that passed through each node. After detection, it alerted the security cloud services to analyze and update their system. This solution achieved the highest classification accuracy of 99.34% with the lowest FPR while detecting compromised IoT devices at distributed fog nodes. Using open sources of distributed computing to distribute the model in fog nodes to detect IoT attack networks and using n-fold cross validation to evaluate performance metrics of design are some of the researchers’ future goals.

#### 3.1.8. Malicious Traffic Classification

In order to protect organizations and individuals against cyber-attacks, network traffic first needs to be analyzed and classified so that anomaly and malicious attacks can be detected. As the role of malicious traffic classification is very important, many researchers have sought to improve classification techniques using the power of AI. Some studies have focused on anomaly and abnormal traffic. Yang et al. [82] built a model that found hidden abnormal traffic in the network to detect attacks using DL techniques. The dataset used was NetFlow campus information, which is a collection of data gathered by campus routers. For the pre-processing stage, the authors transformed the data into standardized format, and then the RNN algorithm was applied. The proposed model resulted in an accuracy of 98%. For future work, the authors propose to search for more critical features that could help in detecting further cyber-attacks.

Chou et al. [83] used AI algorithms through TensorFlow to train the system by providing it with rules and signatures to distinguish between normal and abnormal traffic behavior. The researchers developed a framework of a DL model on TensorFlow by combining multiple layers of non-linear features and training the system to learn the normal behavior using a forward propagation algorithm on the NSL-KDD dataset. The results were promising, showing high accuracy during testing of up to 97.65% in the detection of probing attacks and 98.99% in the detection of DDoS attacks. In future work, improvements need to be made in the training characteristics in TensorFlow as the present model could not predict user to root (U2R—attacker tries to gain unauthorized access posing as a normal user) and remote to local (R2L—attacker tries to gain unauthorized access by exploiting network vulnerabilities) attacks since the dataset sample was too monotonous, leading to over-learning.

An ensemble deep model to detect and classify anomalies at both the network and host levels was presented by Dutta et al. [84]. The datasets used were IoT-23 [61], LITNET-2020 [85], and NetML-2020 [86] and the DL techniques applied were DNN, long short-term memory (LSTM), and a meta-classifier (i.e., LR). A deep sparse autoencoder (DSAE) was used as the feature engineering technique and a stacking ensemble learning approach was used for classification. After testing on three heterogenous datasets, the researchers concluded that the suggested approach outperformed individual and meta-classifiers such as RF and SVM. In future work, the researchers suggest conducting experiments on more sophisticated datasets and using advanced computational methods to boost processing speed.

Sun et al. [87] built a traffic classification model using DL techniques, focusing on web and peer-to-peer (P2P) traffic. The dataset used to train the proposed model was collected by the authors by capturing traffic from the network using a distributed host-based traffic collection platform (DHTCP). In the training process, the dataset was divided by 5:5, 7:3, and 10-fold cross-validation for the first, second, and third experiment, respectively, and radial basis function neural network (RBFNN), SVM, and probabilistic neural network (PNN) were applied. The results showed that the highest accuracy was 88.18% when using PNN and dividing the dataset as 7:3 for training and testing.

Some researchers have focused on investigating the effects of network data representation on the intelligent models. Millar et al. [88] devised and compared three ways of network data representation for malicious traffic classification to deep learners: payload data, flow image, and flow statistics. They showed that malicious classes can be predicted using just 50 bytes of a packet’s payload. Since DL benefits from an extensive and large dataset, the UNSW-NB15 dataset was selected for the experiment. The payload-based method was found to have the best performance. However, all methods failed to accurately identify DDOS attacks. Since different malicious attacks exhibit different defining characteristics, there is no ‘one size fits all’ solution for identifying all attacks. Hence, in future work, the researchers propose to research the combination of payload-based and statistical inputs to identify malicious traffic.

Yang et al. [89] aimed to develop a model for malicious traffic detection of an encrypted network using DL. The model proposed was developed based on a residual neural network (ResNet), which can automatically identify features and effectively isolate contextual information of the encrypted traffic. Moreover, the CTU-13 dataset was used to train the model and, in the pre-processing stage, the data were converted into the IDX format, then traffic refinement, traffic purification, data length unification, and IDX file generation were performed. Then, deep Q-network (DQN) reinforcement learning, and deep convolution generative adversarial networks (DCGAN) were used to generate the encrypted traffic adversarial sample. This resolved the issue of unbalanced and insufficient or small samples. The model achieved a high accuracy of 99.94%. In future, the researchers will focus on delivering advanced genetic algorithms into DCGAN to enhance generator efficiency.

A new framework using ML for hardware-assisted malware detection by monitoring and memory access pattern classification was introduced by Xu et al. [90]. They proposed in-processor monitoring to obtain virtual address trace and addressed this by dividing accesses into epochs and summarizing the memory access patterns of each epoch into features, after which they are fed to ML classifiers, namely RF and LR. It was concluded that the best performing classifier was RF for both kernel rootkits and memory corruption attacks. Its accuracy in kernel rootkits detection reached a 100% TPR, with less than 1% FPR. As for user-level memory corruption attacks, the algorithm demonstrated a 99.0% DR with less than 5% FPR.

De Lucia et al. [91] proposed a malicious network traffic detection mechanism of encrypted traffic using two techniques—SVM and CNN. To conduct the experiments, the team leveraged a public dataset [92], which consisted of malicious and normal TLS network traffic packets. In data pre-processing, the desired TLS features were extracted from the packet captures using a custom program written in the PcapPlusPlus framework [93]. The train–test split was 70–30%. Both methods successfully achieved a high F-score and accuracy and a low FPR. However, SVM outperformed CNN by achieving a lower FPR and a slightly higher F-score, precision, accuracy, and recall.

While building ML models for the detection of normal or malicious traffic, it was observed that questions arise regarding the selection of the right features. With this in mind, Shafiq et al. [94] proposed a ML algorithm called weighted mutual information_ area under the curve (WMI_AUC), a hybrid feature selection algorithm, that helps in selecting the effective features in the traffic flow. The databases used in the study were the HIT Trace 1, which was captured by the authors from WeChat messenger using Wireshark, and the NIMS dataset, which was collected by the authors from their research-tested network. To build the final model, the researchers used 11 different ML algorithms. The model built using the partial decision tree (PART) algorithm resulted in an accuracy of 97.88% using the HIT Trace 1 dataset. For the NIMS dataset, RF resulted in an accuracy of 100%.

Another field that was also covered by researchers was the detection of malicious virtual private network (VPN) traffic. Miller et al. [95] proposed a computational model to address the current limitations in detecting VPN traffic and aid in the detection of VPN technologies that are being used to hide an attacker’s identity. A model was built to detect VPN usage by using a MLP trained neural network by flow statistics found in the captured network packets’ TCP header. The experiment using OpenVPN was able to identify VPN traffic with an accuracy of 93.71% and identify Stunnel OpenVPN with an accuracy of 97.82% when using 10-fold cross-validation. Future studies could be carried out to detect unauthorized user access and research organizational security, which is essential for a business.

Since the spread of malicious websites, research emphasis has been on factor analysis of the site category and the correct identification of unlabeled data in order to distinguish between benign and dangerous websites to mitigate the risk of malicious websites. Wang et al. [96] demonstrated the use of the NB model to classify malicious websites. A self-learning system was developed to categorize websites based on their features, with NB being used to divide the websites into two categories: malicious or benign. The dataset used was the ISCX2016 [97] dataset, which contains over 100,000 URLs and 50 features for each URL. A higher accuracy of up to 90% was achieved after applying factor identification of datasets and accomplishing website classification using the NB classifier, demonstrating that the NB classifier can perform well when it comes to website classification.

Finally, Ongun et al. [98] used the CTU-13 dataset to build ensemble models for malicious traffic detection. The algorithms used to build the model were LR, RF, and gradient boosting (GB). The first representation was connection-level representation where the features were extracted from the raw connection logs. The second representation was aggregated traffic statistics where the authors compared between raw features in the first representation and the features obtained by time aggregation in this representation. The last representation was temporal features, where the authors considered the time interval with the features obtained by time aggregation in the second representation. The best performance achieved by the model built using RF and GB and resulted in high AUC of 99% when applying it on the features of the third representation.

#### Malicious Traffic in a Cloud Environment

Using a dataset constructed from a real cloud environment, Alshammari and Aldribi [99] built ML models to detect malicious traffic in cloud computing. The dataset used was the new ISOT CID [100], a publicly available cloud-specific dataset where the training data contained 17,296 instances and testing had 7411 instances. Their aim was to add some significant features, prepare the training data, and test the dataset against different ML models, namely DT, KNN, NNet, SVM, NB, and RF. The dataset contained 89,364 instances among which 44,569 were malicious and 44,795 were normal instances. They performed both cross-validation (5-, 10-, 15-folds) and split–validation (90–10%, 80–20%, 70–30%). For cross-validation (all 5-, 10-, 15-folds), DT, RF, and KNN all obtained an accuracy of 100%. In the case of split validation (for all 90%, 80%, and 70% splitting), both DT and RF achieved an accuracy of 100%.

Using the same cloud dataset, Sethi et al. [101] proposed an IDS to protect cloud networks from cyber-attacks. The algorithm applied was double deep Q-learning (DDQN). The datasets used were the ISOT CID dataset, and the standard NSL-KDD dataset. The total size of ISOT is 8 TB, but for the purposes of the experiment, only the network traffic data portion was used. For the feature selection phase, the team applied a chi-square feature selection algorithm. The selected features were 164 and 36 for ISOT CID and NSL-KDD, respectively. The accuracy for the proposed model tested for NSL-KDD was 83.40%, whereas for ISOT CID, it was 96.87%. After measuring the robustness of their model against an adversarial attack, the accuracy obtained was 79.77% for NSL-KDD and 92.17% for ISOT CID.

Xie et al. [102] used a class SVM technique based on a short sequence model. They used the Australian Defense Force Academy (ADFA) dataset [103], which contains thousands of normal traces taken from a host setup to simulate a modern Linux server as well as hundreds of anomalous traces caused by six different types of cyber-attacks. As it was a short sequence, duplicate entries were removed, leading to an improved separability between the normal and abnormal. The k values chosen for this experiment were k = 3, 5, 8, 10, with k = 5 providing the greatest results and an accuracy of 70% attained at an FPR of roughly 20%. Although the experimental result showed a significant reduction in computing cost, the rate of an individual kind of attack mode recognition was low.

Vanhoenshoven et al. [104] addressed a variety of ML approaches to solve the challenge of detecting malicious URLs as a binary classification problem including multi-layer perceptron, DT, RF, and KNN. The researchers used Ma et al.’s dataset [105], called the Malicious URLs Dataset, which consists of 121 sets gathered over 121 days. There are 2.3 million URLs and 3.2 million features in the overall dataset. The researchers divided the URLs into three groups based on their characteristics. Each of the methods was used to classify these sets. The models were assessed based on their accuracy, precision, and recall, with features such as blacklists and WHOIS information taken into account. The article implies that all of its approaches achieved high accuracy, with RF being the most convenient approach to use, obtaining an accuracy of roughly 97% based on experimental results. The method also had great precision and recall, demonstrating its reliability.

For the purpose of detecting harmful URLs, Yuan et al. [106] introduced a parallel neural joint model approach. The semantic and text features were included in the method by integrating a parallel joint neural network incorporating capsule network (CapsNet) and independent RNN (IndRNN) to improve the detection accuracy. The malicious URLs data were gathered from two sources: an anti-phishing website called PhishTank and a malware domain list that collects a blacklist of harmful websites. The 5-fold cross-validation technique was applied and unified performance metrics were used to evaluate the model’s performance. According to the results of the experiments, the model performed best when the dimension of the feature was 185 and the number of IndRNN layers was 2. The accuracy and recall rates both reached 99.78% and 99.98%, respectively, resulting in a performance that exceeded traditional models.

By utilizing ML on the latest and more advanced dataset for IoT networks called IoTID 20 [107], Maniriho et al. [108] proposed an approach for anomaly-based intrusion detection in IoT networks. The ML algorithm applied was RF. The dataset had three subsets: subset 1 contained normal and DoS instances; subset 2 contained normal and man-in-the-middle (MITM), and subset 3 contained normal and scan traffic. A 10-fold cross-validation and train–test split of 70–30% were applied. The overall accuracy for each subset attack was DoS—99.95%, Scan—99.96%, and MITM—99.9761% using cross-validation while using the percentage split DoS—99.94%, Scan—99.93%, and MITM—99.9647.

Since the security of IoT networks is a major concern for researchers and decision-makers, some other researchers have used the same IoTID 20 dataset in order to build an IDS for in-home devices. A three-stage strategy that includes clustering with oversampling, reduction, and classification using a single hidden layer feed-forward neural network (SLFN) was provided by Qaddoura et al. [109]. The paper’s significance lies in the data reduction and oversampling techniques used to provide relevant and balanced training data as well as the hybrid combination of supervised and unsupervised techniques for identifying intrusion activities. With a ratio of 0.9 and a k value of 3 for the k-means++ clustering technique, the results showed that using the SLFN classification technique and using the SVM and synthetic minority oversampling technique (SVM-SMOTE) yielded more accurate results than using other values and classification techniques. Similarly, a deep multi-layer classification strategy was suggested by Quddoura et al. [110], which consisted of two phases of detection. The first phase entails detecting the presence of an intrusion and the second phase identifies the kind of intrusion. In preprocessing, the oversampling technique was carried out to enhance classification results. Furthermore, the most optimal model was built, which contained 150 neurons for the single-hidden layer feed-forward neural network (SLFN) (phase 1), and 150 neurons and two layers for LSTM (phase 2). When the findings were compared to well-known classification approaches, the suggested model outscored them by 78% with regard to the G-mean.

#### 3.1.9. Attacks at DNS Level

In order to improve the user’s privacy, a new protocol called DNS over HTTP (DoH) was recently created. This protocol can be used instead of traditional DNS for domain name translation with the benefit of encryption. However, security tools depend on readable information from DNS to detect attacks such as malware and botnet. Hence, Singh and Roy [111] aimed to use ML algorithms to detect malicious DoH traffic. The five ML algorithms used were GB, NB, RF, KNN, and LR. The team conducted the experiment on the benchmark MoH dataset—CIRA-CIC-DoHBrw-2020, which was recently developed and shared publicly [112]. It contained a benign file that had 19,807 instances and a malicious file that had 249,836 instances. The DoHMeter tool [113], which was developed in Python and is freely available, was used to extract important features from the PCAP files. To build the model, the data were split into a train–test ratio of 70–30%. The experimental results showed that RF and GB attained the maximum accuracy of 100%.

#### 3.1.10. Intrusion Detection

NIDS analyzes and monitors the whole network to detect malicious traffic. The following studies used the NSL-KDD dataset. Al-Qatf et al. [114] proposed self-taught learning (STL)-IDS using the DL approach in an unsupervised manner as a feature selection technique to reduce the testing and training time and effectively enhance the accuracy of the prediction for the SVM model. In the pre-processing phase, a 1-n encoding system was applied before STL. Max–min normalization was used to map all features into a specific range. The results obtained through the proposed model represented the classification accuracy of improved SVM compared with algorithms such as J.48, NB, and RF. Moreover, it performed well in five-category (normal and five types of attacks) and two-category (attacks and normal traffic) classification. 

Similarly, to develop a flexible and efficient NIDS, Niyaz et al. [115] proposed a self-taught learning (STL) based on sparse autoencoder (AE) and soft-max regression (SMR) on the NSL-KDD dataset. The authors applied 10-fold cross validation on the training data for STL and applied the dataset directly for SMR. The results showed a high-performance accuracy rate of 98% for STL.

Following the same principle of using DL for intrusion detection, Zhang et al. [116] proposed an approach using the NSL-KDD dataset, consisting of normal and different forms of abnormal traffic. By first applying feature selection to remove the unrelated features and noise, the autoencoder was implemented to learn the features of the input data and extract the key features. Soft-max regression classification was then applied. The measures for evaluation used were accuracy, precision, recall, and F-score. Finally, the model achieved F-score and recall values of 76.47% and 79.47%, respectively.

Some studies have focused on multi-layer DL algorithms. Wu and Guo [117] proposed a LuNet model, which is a hierarchical CNN and RNN neural network, applied on the NSL-KDD and UNSW-NB15 dataset. They started by converting the categorical features using the ‘get dummies’ function in Pandas, then they applied standardization to scale input data and concluded by employing K-fold cross-validation. To evaluate LuNet, the following evaluation criteria were used: accuracy, FPR, and DR. The performance in binary classification achieved on average 99.24% on the NSL-KDD dataset and 97.40% accuracy on the UNSW-NB15 dataset. The performance in multiclass classification was an average of 99.05% accuracy on NSL-KDD, and 84.98% accuracy on UNSW-NB15. In future work, the researchers intend to investigate worms and backdoors as these were wrongly classified in the model.

To detect network intrusions efficiently, Hasan et al. [118] used an ANN. Different backpropagation algorithm training approaches were employed to detect the attacks and non-attack connections. The DARPA 1998 [119] intrusion detection dataset was used for training and testing purposes. To train the model, the researchers used the backpropagation learning algorithm, letting it detect intrusions in the following three modes: batch gradient descent with momentum (BGDM), batch gradient descent (BGD), and resilient backpropagation (RP). Finally, they used the DR and the FPR to determine the performance of intrusion detection. The total attack detection performance and the efficiency measure support the RP method of training, which obtained an accuracy of 92%. Further changes in the network architecture can be made to enable the efficient use of the network with other approaches.

Likewise, Devikrishna et al. [120] proposed an approach that used ANN as a pattern recognition technique to classify normal and attack patterns. The dataset used was the KDD-99 dataset. The feature extraction process consisted of feature selection and feature construction. An MLP was used for intrusion detection. MLP was a layered feed-forward ANN network typically trained with backpropagation. Accuracy was a goal that largely improved the overall effectiveness of the IDS. A possible future research direction could be to incorporate more attack scenarios in the dataset.

Abuadlla et al. [121] also proposed an IDS based on flow data built in two stages. The first stage involved the detection of abnormal traffic on the network. The second stage involved detecting and classifying the attack types in the network traffic. The NetFlow dataset made by network captures was employed to train the proposed system. To build the proposed model, a multilayer feedforward neural network and the radial basis function network (RBFN) were used. The proposed model resulted in a higher accuracy of 94.2% for the abnormal traffic detection stage, and 99.4% for the attack detection and classification stage. Although the multilayer feedforward neural network resulted in higher accuracy, it consumed more time and memory in comparison with RBFN, which makes RBFN a better choice for real-time detection. In future work, the researchers aim to build a faster and more accurate model for real-time detection with a smaller number of features.

Utilizing the KDD-99 dataset, Alrawashdeh et al. [122] aimed to build a DL model for anomaly detection in real-time. The researchers began by transforming categorical features into numerical features for convenience. Then, they removed the duplicated records to reduce computational time and improve performance. Three models were built: first using the restricted Boltzmann machine (RBM), the second using deep belief network (DBN), and the third using DBN with LR. The model that was built using DBN and LR resulted in the best performance with an accuracy of 97.9% and a FN rate of 2.47%.

In addition, Al-Janabi et al. [123] proposed a model based on ANN using the KDD-99 dataset and incorporated three scenarios: detection mode, detection and classification mode, and detailed classification mode. The researchers performed their experiment for each scenario by training the models using different number of features in each. The best results achieved were a 91% DR and 3% FP rate using 44 features with the detection only scenario. The results showed that performance decreased as a higher level of classification was performed.

Belavagi et al. [124] evaluated the different ML algorithms used to classify the network data traffic as normal traffic or intrusive (malicious) traffic. By using the NSL-KDD dataset consisting of internet traffic record data, supervised ML classifiers, namely LR, SVM, Gaussian NB, and RF were applied to identify four simulated attacks. After converting all the categorical data to numerical form in the pre-processing stage, the predicted labels from these models were compared with the actual labels, and TPR and FPR were computed. From the observed results, it was concluded that the RF classifier outperformed other classifiers for the considered dataset, with an accuracy of 99%. The researchers suggested that the work can be further extended by considering the classifiers for multiclass classification and considering only the important attributes for intrusion detection.

Additionally, Almseidin et al. [125] evaluated the different ML algorithms, keeping the focus on FNR (identifying an attack as normal traffic) and FPR (identifying normal traffic as an attack) performance metrics to improve the DR of the IDS. They used several algorithms, namely J.48, RF, random tree, decision table, multi-layer perception (MLP), NB, and Bayes network. The KDD-99 dataset was imported to SQL server 2008 to implement statistical measurement values such as attack types and occurrence ratios. Then, 148,753 record instances were extracted for training data. A wide range of results was obtained by using Weka tools that demonstrated that the RF achieved the highest average accuracy and the decision table achieved the lowest FNR.

Choudhury et al. [126] implemented ML algorithms to categorize network traffic as normal or anomalous. Algorithms such as BayesNet, LR, instance-based knowledge (IBK), J.48, PART, JRip, random tree, RF, REPTree, boosting, bagging, and blending were incorporated and compared. The researchers used the NSL-KDD dataset and Weka tools to model and compare the algorithms. The results showed that RF achieved the highest accuracy of 91.523%, and the lowest accuracy of 84.96% resulted from LR.

Similarly, the objective of the system proposed by Thaseen et al. [127] was to detect any intrusions in the network using ML by classifying different packets without decrypting their content. For intrusion detection analysis, packets were generated and transmitted over a network and were captured by Wireshark. This captured data was organized into a dataset. By implementing ML algorithms such as NB, SVM, RF, and KNN, the data were classified with an accuracy of 83.63%, 98.23%, 99.81%, and 95.13%, respectively. Future work to this study includes the plan to use DL algorithms to enhance the performance and accuracy of recognition and classifying different types of packets transmitted over a network.

Likewise, Churcher et al. [128] proposed several ML models to cope with the increase in the number of network attacks. The researchers highlighted several ML methods that were used in IDS such as DT, SVM, NB, RF, KNN, LR, and ANN. The Bot-IoT dataset [129] containing ten CSV files that have records of IoT network attacks and 35 features was used. In the pre-processing stage, the undesirable features were removed. The results of the model showed that in RF, the accuracy for DDoS attacks was 99% in binary classification and its performance was superior in the context of all types of attacks. However, KNN achieved 99% accuracy and outperformed other ML algorithms in the multiclass classification. In conclusion, KNN and ANN are more accurate when used in weighted and non-weighted datasets, respectively, for multiclass classification.

A comparative analysis of two commonly used classification methods, SVM and NB, to evaluate the accuracy and misclassification rate was conducted by Halimaa et al. [130] using the NSL-KDD dataset. For comparative analysis, the Weka tool’s randomized filter was used to ensure the random selection of 19,000 cases. The results showed that SVM attained an accuracy of 93.95% and NB achieved an accuracy of 56.54%. The researchers plan to work with larger amounts of data and construct a cross multistage model to create the ability to categorize additional attacks with accuracy and better performance.

Ghanem et al. [131] assessed the performance of their existing IDS against 1- and 2-class SVMs by applying both straight and non-linear forms. For the first step of data collection, they collected five datasets from the IEEE 802.11 network testbed and another dataset was collected in Loughborough University from an ethernet local area network office. All this traffic was collected in the PCAP structure using tcpdump. The results demonstrated that the linear 2-class SVM presented generally highly accurate findings. In addition to reaching a 100% success rate over four out of five of the metrics, it required training datasets. Meanwhile, the linear 1-class SVM’s performance was nearly as good as the best technique and did not require training the dataset. Overall, it was concluded that the existing unsupervised anomaly-based IDS can benefit from using any of the two ML techniques to improve accuracy in detection and its analysis of traffic, especially when it is comprised of non-homogeneous features.

Mehmood et al. [132] focused on supervised learning algorithms to make a comparison of three ML algorithms, namely SVM, J.48, NB, and decision table for anomaly-based detection. These algorithms were trained using the short version of the KDD-99 dataset as it has many records. The performance measures used in this comparison were FPR, TPR, and precision. The results highlighted a limitation when it came to DR, as not a single algorithm had a high DR for all the tested attacks in the KDD-99 dataset. However, the J.48 had a low misclassification rate. Hence, it was concluded that this algorithm performed best out of all the other algorithms.

An approach that boosts the capacities of wireless network IDS was introduced by AlSubaie et al. [133]. The dataset used was WSN-DS [134], which included 23 attributes and five potential outputs (four attacks (DoS attack): flooding, grayhole, blackhole, and scheduling and one normal state (no attack)). The ML algorithms used here were ANN and the J.48. Additionally, the data noise was calculated as it affects the accuracy of the ML algorithms. The amount of noise permissible for the ML model to be deemed trustworthy was determined. The results determined that J.48 performed better than the ANN when noise was not considered, obtaining the highest accuracy rate of 99.66%. With datasets having more noise, ANN was more tolerable.

In order to determine which of the models could handle large amounts of data and still produce accurate predictions, Ahmad et al. [135] used the SVM linear and radial basis function (RBF), RF, and ELM methods and compared their performance on the NSL-KDD dataset. The results demonstrated that when using the full dataset, the ELM outperformed the other algorithms in terms of all the metrics being tested in all experiments including accuracy, which reached 99.5%. On the other hand, when using half and a quarter of the dataset, SVM performed better overall, with an accuracy of around 98.5%. Hence, it was concluded that ELM is best suited for intrusion detection when dealing with large amounts of data. The researchers plan to further explore ELM and experiment with it using different selection and feature transformation techniques and their impact on its performance.

Amira et al. [136] found MLP to be the most effective and appropriate classifier to increase detection accuracy. The data pre-processing phase was carried out using the equal width binning algorithm. The sequential floating forward selection (SFFS) feature selection technique was applied, resulting in the selection of 26 features. Using the NSL-KDD dataset, Amira et al. then applied a multi-agent, 2-layer classification algorithm. The different classifiers that were tested and compared were: NB and DT, namely NBTree, BFTree, J.48, and RF Tree. NBTree and BFTree gave better results than RF and J.48. MLP gave good results in terms of classifying normal and DoS attacks compared to identifying the R2L and U2R attacks. Overall, it was concluded that a single classifier is not sufficient to classify the attack class. Therefore, to increase classification accuracy, multiple classifiers must be involved.

Rather than comparing different techniques, Gogoi et al. [137] focused on evaluating the clustering approach to detect network traffic anomalies on different datasets. The proposed method was evaluated using TUIDS [138] datasets, the NSL-KDD dataset, and the KDD-99 datasets. The real-life TUIDS intrusion datasets consist of three datasets: flow level, packet level, and port scan. After the pre-processing stage, they applied a combination of supervised clusters and unsupervised incremental clusters which labelled the training data into different profiles (or rules). The prediction was undertaken using a supervised classification algorithm. Using the TUIDS dataset, the packet level had the highest accuracy of 99.42%. When using the KDD-99 dataset, the accuracy achieved was 92.39%. Finally, using NSL-KDD, the accuracy achieved was 98.34%.

Aiming to classify real-time traffic by using 12 features of network traffic data to classify 17 attack types of DoS, probing as well as normal was conducted by Wattanapongsakorn et al. [139]. Supervised ML techniques—DT, ripple rule, back-propagation neural network, and Bayesian network—were applied. In the pre-processing stage, the team used a packet sniffer and a built-in Jpcap library to collect and store network records over a period of time. Then, in the classification part, training and testing were performed using Weka tool, and results were observed. The DT approach achieved the highest DR of 85.7%. In the second experiment, some attack types were grouped together, and training data consisted of 9000 records with 600 records of each attack type (so 600 × 15). In this case, the DR was much higher, with the DT being 95.5%.

Further research that worked on enhancing an existing algorithm for intrusion detection was done by Cui et al. [140], who worked on enhancing the Bayes classifier (BC). The proposed method seeks to integrate the spatiotemporal patterns of measurement into a flexible BC to detect cyber-attacks. Spatiotemporal patterns were captured by the graph Laplacian matrix for system measurements. After the evaluation of the developed method’s performance, it was concluded that the flexible BC showed the largest TPR compared with the naïve BC, SVM, and DT methods, which verified the effectiveness of the developed method. For future work, DL techniques will be involved by mapping the spatiotemporal patterns to a linear space using the LSTM network for better detection accuracy of cyber-attacks.

Moreover, Kumar et al. [141] focused on enhancing the detection efficiency by combining three algorithms—RF, JRIP, PART—to identify threats of mobile devices. The dataset used contained around 600 samples that were captured by the researchers from the virtual machine using Wireshark. For feature extraction, the researchers used bidirectional flow export using the IP flow information export method (RFC-5103 BiFlow). The challenge the researchers faced was an overfitting problem and concept drift condition, which is caused by choosing low performance giving features. The ensemble model resulted in an accuracy of 98.2% with the ability to identify benign traffic. For future work, the researchers aim to integrate ML with conventional NIDS and to reduce the chance of concept drift by introducing innovative methods.

Similarly, Tahir et al. [142] constructed a hybrid ML technique for detecting network traffic as normal or intrusive by combining K-means clustering and SVM classification to improve the DR and to reduce the FPR alarm and FNR alarm. The dataset applied in the proposed technique was the NSL-KDD dataset. Pre-processing was performed on the dataset to reduce ambiguity and supply accurate information to the detection engine. After applying the classifier subset evaluator and best-first search algorithms, both the classifiers—K-means and SVM—were then tested and their performance evaluated. The hybrid ML technique results showed that they attained 96.26% as the DR and 3.7% as the FNR. The model showed a comparatively higher detection for DoS, PROBE, and R2L attacks.

One more enhanced technique was proposed by Sharma et al. [143] to apply efficient data mining algorithms for detecting network traffic as normal or anomalous. The team applied KDD-99, which contains 4.9 M data instances and four class types. In feature selection, they collected basic features such as protocol type, duration, flags, etc. The data was normalized and the classification was carried out using k-means clustering via a NB classifier. The target variable was classified as normal, DoS, U2L, R2L, probing. The DR achieved by using the proposed method was 99%.

Following the same ideology, Lehnert et al. [144] built their system in steps with more complexity added at each level. They used the KDD-99 dataset and Shogun ML Toolbox to test and train the data. The study’s focus was mainly on using the SVM implementation provided by the toolbox. The key step in this paper was the training phase, which was done using labelled data. The goal was to attempt to choose the most appropriate kernel and minimize the number of features. The results showed that two out of the four available kernels on Shogun tied in the best accuracy. These kernels were Gaussian and Sigmoid, which produced an error of only 2.79%. It was concluded that identifying both the kernel that has the lowest error rate and the subset of the most relevant features leads to an improved version of the algorithm. Ultimately, this can enhance the accuracy and efficiency of the SVM applied for intrusion detection, making it able to predict with higher speed and accuracy.

An innovative feature selection algorithm called the ‘highest wins (HW)’ was proposed by Mohammad and Alsmadi [145] in order to enhance intrusion detection. This HW algorithm was applied in NB techniques on 10 benchmark datasets from the UCI repository to evaluate its performance. The results showed that the proposed HW algorithm could successfully reduce the dimensionality for most of these datasets compared to other feature selection methods such as chi-square and IG. The team conducted another set of experiments where NB and DT (C4.5) classifiers were built using the HW technique on the NSL-KDD dataset on its binary and multiclass versions. For binary, HW reduced the features of the dataset from 41 to eight and the results gave an accuracy of 99.33% using the reduced features (0.23% decrease compared to using complete features). For multiclass, HW reduced the features of the dataset from 41 to 11, and in terms of time needed for building the model, reduced features had an enhancement of 2.3%. The results demonstrated that instead of using all 41 features of this dataset, using only eight by applying HW could produce classifiers with the same classification performance.

Furthermore, Chawla et al. [146] proposed a computational efficient anomaly-based IDS that was a combination of CNN and RNN. To detect malicious system calls, they merged stacked CNNs with GRUs. Using the ADFA dataset of system call traces, they obtained a set of equivalent findings with shorter training periods when using GRU. They employed CNN to extract the local features of system call sequences and feed them into the RNN layer, which was then processed through a fully connected SoftMax layer, which generates a probability distribution across the system calls processed by the network. Trained on normal system calls, which predict the likelihood of a subsequent system call, a testing sequence was employed to detect a malicious trace based on a pre-defined threshold. The RNN-based LSTM model’s training time was claimed to be reduced by the researchers.

In addition, Nguyen et al. [147] used the DL approach for detecting cyber-attacks in a mobile cloud environment. The used datasets were KDD-99, NSL-KDD, and UNSW-NB15 (training = 173,340 records, testing = 82,331 records). The researchers adopted principal component analysis (PCA) to reduce the dimensions for the datasets and the learning process comprised of three layers: the input layer, hidden layers, and output layer. The input layer used Gaussian restricted Boltzmann machine (GRBM) to transform real values to binary code. The hidden layer used restricted Boltzmann machine (RBM) to perform the learning process. The output of the hidden layer was used as input in the output layer (SoftMax regression step). They used accuracy, recall, and precision for measuring performance. The results showed that the accuracy for NSL-KDD, UNSW-NB15, and KDD-99 datasets, respectively, were 90.99%, 95.84%, and 97.11%. For future work, Nguyen et al. proposes implementing the model on real devices to measure the accuracy on a real-time basis and evaluate the energy and time consumed in the detection.

An improved IDS was proposed by Tama et al. [148] where they used two datasets to evaluate the performance of the model: NSL-KDD and UNSW-NB15. To minimize the feature size, a hybrid feature selection technique was used. The hybrid feature selection consisted of three methods: the ant colony algorithm, particle swarm optimization, and genetic algorithm. Then, the researchers proposed a two-stage classifier ensemble, which was rotation forest and bagging. The proposed model achieved an accuracy of 85.8% with the NSL-KDD dataset and 91.27% with the UNSW-NB15 dataset. For future work, the researchers intend to perform the proposed model to solve the multiclass classification problem.

A novel intrusion detection system was proposed that takes the advantage of both statistical features and payload features by Min et al. [149]. They used the ISCX2012 dataset, which is more updated and closer to reality, and they utilized word embedding and text-CNN to extract more features from the payloads. Then, the RF algorithm was applied on the combination of payload features and statistical features, where they named the model with TR-IDS. Moreover, the effectiveness of TR-IDS was compared against five ML models, namely SVM, NN, CNN, and RF (RF-1) and RF (RF-2, which used statistical features only). The highest result achieved was by TR-IDS with an accuracy of 99.13%.

Finally, more information on intrusion detection using unsupervised and hybrid methods can be found in a survey paper composed by Nisioti et al. [150]. They presented and highlighted important issues such as feature engineering methods for IDS. Furthermore, using IDS data to construct and correlate attacks to identify attackers as well as extending the current IDS to identify modern attacks were all addressed by the paper.

Table 2 below presents a summary of all details discussed in this section, giving overview picture of all reviewed articles in terms of research problem domain targeted, dataset used, and intelligent techniques applied as well as the results achieved.

### 3.2. Common Intelligent Algorithms Applied

**Figure 1:**
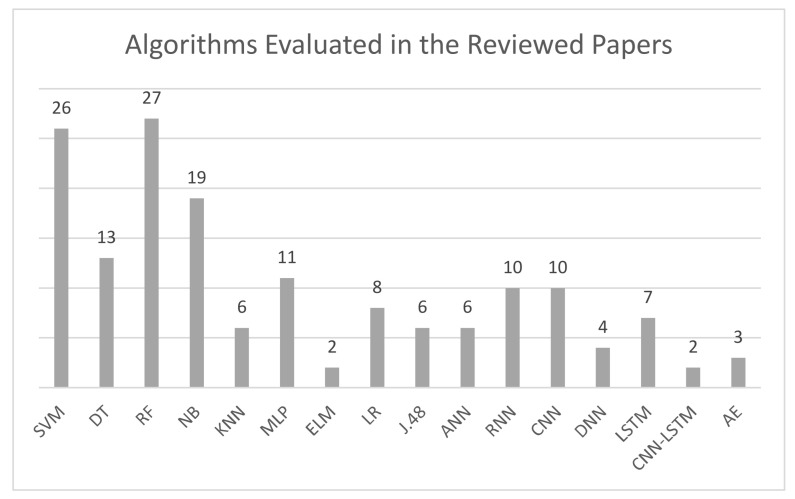
ML and DL algorithms used in the reviewed papers.

In this literature review, a number of papers were studied between the period of 2010–2021 and a plethora of both ML and DL techniques were utilized in these papers to build or compare models to detect and classify network attacks. Table 3 presents a list of all the respected papers that utilized the different algorithms, highlighting all problem domains where each algorithm was used for as well as the highest performance achieved. Figure 1 presents the number of articles that utilized each algorithm. As seen from the figure and table, RF and SVM were the most widely used algorithms in a good number of papers and ELM was the least applied algorithm. For ML algorithms, the best performing algorithms were DT, RF, and KNN with their accuracy reaching up to 100% and the least utilized algorithms were J.48 and KNN. For DL algorithms, the best performing algorithm was RRN with the highest accuracy of 100% achieved and the least utilized and least popular algorithm was ELM, which is considered to be fast in terms of training as it consists of a single hidden layer, so it is usually applied to simple applications. However, it has recently been extended to be hierarchical to handle more complex problems with higher accuracy [152].

### 3.3. Common Datasets Used

There are several datasets used by researchers in the reviewed papers to evaluate their network detection and classification model. The most widely used dataset is NSL-KDD due to the reasonable size of its training and testing sets and is also available publicly. There are 41 features in the NSL-KDD dataset. It is an enhanced version of the KDD dataset and removed the duplication of the records to eliminate the bias of the classifiers. Then, KDD-99 and CICIDS2017 came after NSL-KDD. The KDD-99 dataset was used for the first time in a competition and is an improved version of DARAP98. The CICIDS2017 dataset contains normal and new attacks and was published in 2017 by the Canadian Institute for Cybersecurity (CIC).

After that, the UNSW-NB15 dataset comes next in terms of repeatedly being used. The IXIA tool was used for creating the UNSW-NB15 dataset and it consists of nine types of attacks.

There are many other datasets, however, few researchers have tried to create their datasets. The CTU-13 dataset was captured by CTU University in the Czech Republic. It contains real botnet traffic combined with normal traffic and contains thirteen scenarios including legitimate traffic and attacks such as DoS. The SNMP-MIB dataset consists of about 4998 records with 34 variables. The attacks recorded in the data include six DoS attacks (TCP-SYN, ICMP-ECHO, HTTP flood, UDP flood, Slowloris, Slowpost) and web brute force attacks. The Kyoto 2006+ dataset was built from real traffic data from Kyoto University’s Honeypots over three years, from November 2006 to August 2009. The Kyoto 2006+ dataset consists of 24 features, 14 of which are derived from the KDD-99 dataset and 10 additional features that can be used to analyze and evaluate the IDS network. Honeypots, email server, darknet sensors, and web crawler were used to construct the Kyoto 2006+.

ADFA is an IDS that includes three data types in its structure: (1) normal training data with 4373 traces; (2) normal validation data with 833 traces; and (3) attack data with 10 attacks per vector. As the web became a significant internet criminal activity platform, the security community put in efforts to blacklist malicious URLs. Ma et al.’s dataset [153] consists of 121 sets with overall 2.3 million URLs and 3.2 million features in the dataset. The researchers divided the URLs into three groups based on their characteristics, with features being identified as binary, non-binary, numerical, or discrete.

**Figure 2:**
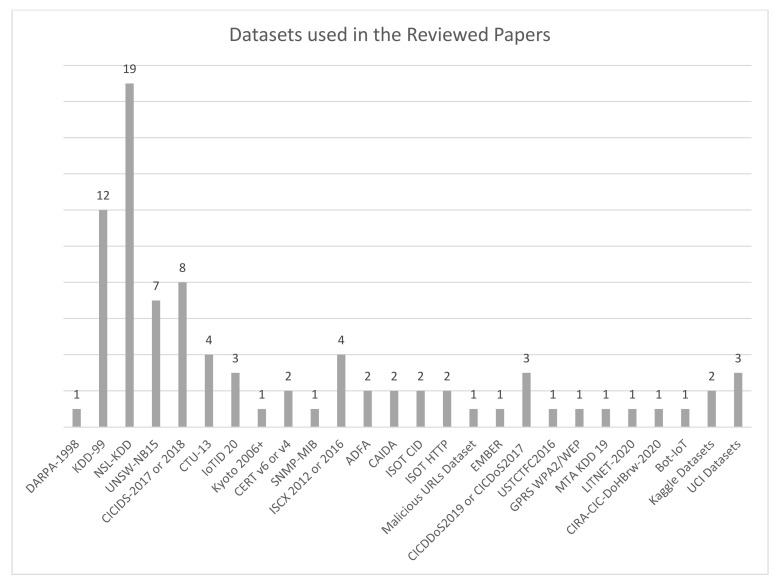
Datasets used in the reviewed papers.

Table 4 lists all the respected papers that utilized the different datasets, highlighting the main references for all datasets as well as the last year when each dataset was used. Figure 2 presents the number of articles that utilized each dataset.

## 4. Discussion and Conclusions

Network security is a major concern for individuals, profit, and non-profit organizations as well as governmental organizations. In fact, with the digital explosion that we are witnessing in the present era, ensuring network security is an urgent necessity in order to safeguard society’s acceptance for thousands and thousands of services that rely essentially on the backbone of the digital life, which is the network. Therefore, network security turns out to be an urgent requirement, and not a luxury. Although many protection methods have been introduced, there are still some vulnerabilities that are exploited by hackers, leaving the network security administrators in a continuous race against the network attackers. Techniques that hover around the use of intelligent methods, namely machine learning (ML) and deep learning (DL) have proved their merits in several domains including health care systems, financial analysis, higher education, energy industry, etc. This indeed motivated the people responsible for the network security to further explore the ability of these techniques in providing the required level of network security. Consequently, several intelligent security techniques have been offered in the past few years. Although these techniques showed exceptional performance, the problem has not been resolved entirely. This leaves us in a position to critically evaluate the currently offered solutions to recognize the possible research directions that might lead to building more secured network environments.

The complication of using the right dataset and features or the right ML and DL algorithms to identify the different attack types has proven to be an arduous decision for experts to make. Hence, among the reviewed papers, some researchers focused on comparing different algorithms to determine which algorithm to use for building an intelligent model using a training dataset. As no algorithm has been found to be a silver bullet for identifying and classifying all attacks with high accuracy, it was widely noted that it is not reasonable to accept a single algorithm as a universal model.

When building any intelligent system, the designer should take into account what is/are the algorithm(s) that best fit the domain. Not only this, but the designer should also decide which dataset comprises a set of features that better represent the classification area. Considering the network attacks, this research article found that RF is the most commonly used algorithm and this can be justified due to the fact that it uses an ensemble learning technique, which to some extent might ensure a life-long system due to the exceptional capability to continuously learn new knowledge on the fly. Producing models with reduced overfitting is another motivation behind using the RF. Not only this, but RF can also be effectively applied on both categorical and continuous features, and thus it can be applied to a wide range of datasets. In addition, the exceptional ability to handle missing data puts RF as a first option when building network attack mitigation models taking into account that most of the datasets are susceptible to include missing values. However, since RF produces complex trees, building a real-life system based on RF could be a challenging task because it might require more computational power and resources, while in fact, the main success factor for building a system for detecting network attacks is the quick and instant reaction. SVM is the second most widely used algorithm. However, SVM is applied to a fewer number of network attacks when compared to RF. This can be justified due to the fact that SVM produces complex intelligent models that are difficult to apply in real life. Nevertheless, SVM is considered as the main competitor to RF due to the fact that it shares several advantages with RF such as the exceptional capability to deal with missing values, and the remarkable capability to reduce the overfitting problem. NB ranks in third place, but still did not achieve the same predictive performance as RF and SVM due to the fact that it assumes that the dataset features are independent, which in fact, is not true in most training datasets. DT was employed almost half the time that RF and SVM were used. DT proved its merits in several domains, but in the network security domains, it has not been used very much. This can be justified due to the fact that it produces a set of rules that if exposed to the attackers, they can adopt their attacks by avoiding the rules adopted from the DT models.

Included among the algorithms that conveyed excellent performing results were DL models, namely, DNN and RNN as well as ML models, namely, RF and DT with their accuracies reaching up to 100%. A more promising research direction to explore can increasingly be toward applying hybrid or ensemble models to improve attack detection accuracy; for instance, augmenting DL techniques such as CNN with long short-term memory (LSTM) for automating feature engineering and improving network attack detection accuracy. Furthermore, gated recurrent unit (GRU), initially proposed in 2014, can further be applied by researchers in solving various problem domains in network security as it is considered more efficient than LSTM, and it uses comparatively less memory, and executes faster. They can solve complex problems faster, if trained well, and therefore, they are worth trying in network attack detection, namely for DDoS or in IoT networks.

Since the performance of the intelligent models largely depend on the datasets used for training them, it is important to analyze and evaluate which dataset to use for which type of attack. It is recommended that large datasets are used with a good distribution of each class type to increase the detection and classification accuracy. Moreover, limited availability of such datasets represents a challenge in the development of more robust intelligent-based models and highlights the need for producing and publishing more new datasets in different network attack problem domains. Most of the authors in the reviewed articles used the KDD-99 dataset as well as its latest version, the NSL-KDD dataset. However, the ADFA dataset was also used by some, which was proposed as a replacement for the KDD-99 dataset, ISOT HTTP for botnet, ISOT CID for cloud environments, and IoT20 for IoT environments, so can be explored further and used to build different ML and DL models.

Identifying malicious and benign URLs was also a fundamental research direction carried out by researchers where an important set of features that affected the model accuracy were URL related features. It was found that additional improvements in classifying malicious and benign URLs can be accomplished by deploying a lexical approach, which uses static lexical features extrapolated from the URL, in addition to analyzing the URL contents for instantaneous and reliable results. Hence, using a lexical approach to classify URLs can be an important direction to explore.

Several other problem domains need to be explored as they could be a valuable direction for enhancing network security in the modern world. Namely, with the growing establishment of encrypted network traffic as well as virtual private networks, more research needs to be carried out in detecting malicious traffic in these domains using intelligent techniques as not enough research has been focused in this area. Furthermore, with the rising number of inter-connected devices and the establishments of Internet of Things (IoTs) networks, more investigation needs to be carried out in assessing different intelligent techniques on new datasets such as IoT20 as well as paving ways to developing software that can detect and analyze data packets communicated in IoT environments to update the existing datasets for more attacks. Additionally, a new protocol called DNS over HTTP (DoH) has been created recently for which more research needs to be explored on detecting malicious DoH traffic at this (DNS) level.

Finally, multiple researchers intend in their future work to convert the models they built into a real-time system in order to benefit from them in real-life scenarios such as in attack detection and prevention. There are two levels of real-time ML which are online predictions and online learning. Online prediction means making predictions in real-time. Furthermore, online learning allows for the system to incorporate new data and update the model in real-time. Hence, converting intelligent models into real time systems may be considered as a fundamental direction to probe by more researchers.

## Figures and Tables

**Table 1:** Types of network attacks.

Attack Name	Description	Attack by (Packets, Tools, etc.)
Active Attacks		
**Denial of Service (DoS) Attacks**		
Jamming Attack	By using the channel that they are communicating on, it prohibits other nodes from accessing it to connect.	Radio frequency noise.
Flooding	A DoS attack in which a server receives many connection requests but does not reply to complete the handshake. (ICMP Flood, SYN Flood, HTTP Flood).	Unbound number of requests without acknowledgment of packet after receiving it.
Smurf Attack	A network layer DDoS attack caused due to the network tools misconfiguration.	Source IP fooling victim IP.
Teardrop Attack	A DoS attack that bombards a network with many Internet Protocol (IP) data fragments, then the network is unable to recombine the fragments back into their original packets.	Sending fragmented packets to the target machine.
**Man in the Middle Attacks**		
Ransomware	A form of malware that infiltrates and encrypts important files and systems, preventing a person from accessing their own data.	B0r0nt0k (encryption ransomware), Mado (malicious program)
Session Hijacking	To obtain unauthorized access to the Web Server, the Session Hijacking attack disrupts the session token by stealing or guessing a valid session token (e.g., predictable session token).	Malicious JavaScript Codes, XSS, Session Sniffing.
**Passive Attacks**		
Active Reconnaissance	An intruder is engaged in targeting the system to acquire information about vulnerabilities (e.g., port scanning).	Nmap, Metasploit.
Passive Reconnaissance	Gathering information about computers and networks without actively engaging with them (e.g., eavesdropping, OS fingerprinting).	Wireshark, Shodan.
Traffic Analysis	A method to gather and monitor wireless frames, packets, or messages to drive information for communication patterns.	Sniffing tools.
War Driving	Mapping the wireless access points with wireless networks with vulnerabilities in moving cars.	iStumbler, Global Positioning System (GPS), antenna, Wifiphisher.
**Bitcoin Attack**		
Zero Access	An attack that has an unknown pattern or aims to exploit a potentially serious software security vulnerability that the developer or security personnel are not aware of.	Undiscovered vulnerabilities (hardest to detect).
**Account Attacks**		
Credential Stuffing	A kind of cyber-attack in which attackers break into a system using a list of compromised user credentials. (e.g., dictionary attack).	Bots for automation, fake IP addresses.
Account Takeover	Account Takeover is like identity theft where a criminal gets unauthorized access to another person’s account (e.g., phishing, call center fraud).	Obtaining compromised credentials.
Account Lockout	An attacker who does not have access to genuine website users’ credentials yet nevertheless does harm to them by taking advantage of security mechanisms (e.g., brute force attack).	Locking a huge number of user accounts.
**Security Breaches**		
Vulnerability Scanning	A continuous automated process of finding security flaws in websites on a network to exploit threaten and attack those websites.	Bots that look for security issues and match them to known vulnerabilities in a database.
API Abuse	API Abuse is defined as unauthorized or unlawful access to a server’s API via mobile or desktop applications.	Stealing application codes for valuable intellectual property.

**Table 2:** Brief summaries of the reviewed papers.

Authors	Year	Problem Domain	Dataset	Techniques	Results (Evaluation Metrics)
Churcher et al. [128]	2021	IDS	Bot-IoT	KNN, SVM, DT, NB, RF, LR, ANN	Binary class: Accuracy (RF-99%) Multi-class: Accuracy (KNN-99%)
Yang et al. [89]	2021	Malicious Traffic	CTU-13	ResNet + DQN + DCGAN	Accuracy-99.94%
Tuor et al. [10]	2021	Insider Threat	CERT v6.2	SVM, isolation forest, DNN, RNN	Recall (DNN, RNN, isolation forest-100%)
Marin et al. [62]	2021	Malware Attack	USTCTFC2016	DeepMAL-using CNN layers	Accuracy (Rbot-99.9%, Neris-63.5%, Virut-54.7%)
Ahuja et al. [24]	2021	DDoS	Private Dataset	CNN, RNN, LSTM, CNN-LSTM, SVC-SOM, SAE-MLP	Accuracy (SAE-MLP-99.75%)
Yuan et al. [106]	2021	Malicious Traffic	Private Dataset	Neural Network, RNN	Accuracy (CapsNet, IndRNN = 99.78%)
Alshammari et al. [99]	2021	Malicious Traffic	ISOT CID	DT, KNN, RF, NB, SVM, NNet	Cross val: Accuracy (RF, DT, KNN-100%) Spit val: Accuracy (RF, DT-100%)
Mohammad and Alsmadi [145]	2021	IDS	NSL-KDD10 UCI benchmark datasets	NB and C4.5 using HW	Reduced features give similar results Accuracy (C4.5-93.90%)
Qaddoura et al. [109]	2021	Common IoT attacks	IoT 20	SLFN	SLFN + SVM-SMOTE: ratio-0.9, k value-3 for k-means++
Qaddoura et al. [110]	2021	Common IoT attacks	IoT 20	LSTM, SLFN	G-mean (LSTM + SLFN-78%)
Maniriho et al. [108]	2021	Common IoT attacks	IoT 20	RF	DoS: Accuracy-99.95% MITM: Accuracy-99.9761% Scan: Accuracy-99.96%
Butnaru et al. [51]	2021	Phishing Attacks	Public Dataset from Kaggle & PhishTank	RF, MLP, SVM, NB, DT	Accuracy (RF-99.29%)
Lin et al. [50]	2021	Phishing Attacks	Private Dataset	Neural Network (Phishpedia)	Accuracy (Phishpedia-99.2%)
Rehman et al. [42]	2021	DDoS	CICDDoS2019	GRU, RNN, NB, SMO	Accuracy (GRU-99.94%)
Wang et al. [96]	2020	Malicious Traffic	ISCX 2016	NB	Accuracy (NB-90%)
Miller et al. [95]	2020	Malicious Traffic	Wireshark Network Captures	Neural Network	Accuracy (NNet-93.71%)
Thaseen et al. [127]	2020	IDS	Wireshark Network Captures	NB, SVM, RF, KNN	Accuracy (RF-99.81%)
Alam et al. [43]	2020	Phishing Attacks	Phishing dataset from Kaggle	RF, DT	Accuracy (RF-97%)
Barut et al. [60]	2020	Malware Traffic	Dataset from Stratosphere IPS, CICIDS2017	NB, C4.5, DT, RF, SVM, AdaBoost	Accuracy, DR (RF-99.996%), FAR (RF-2.97%)
Pande et al. [28]	2020	DDoS	NSL-KDD	RF, SVM, Clustering, Neural Networks	Accuracy (RF-99.76%)
Cui et al. [140]	2020	IDS	Network Captures	BC	TPR (BC-98.75%)
Alsubaie et al. [133]	2020	IDS	WSN-DS	J.48 form of DT, ANN	Accuracy (J.48-99.66%)
Dutta et al. [84]	2020	Malicious Traffic	IoT-23, LITNET-2020, and NetML-2020	ensemble of DNN, LSTM, DSAE	Accuracy-99.7%
Al-Haija et al. [74]	2020	Common IoT attacks	NSL-KDD	CNN	Binary class: Accuracy-99.3% Multiclass: Accuracy-98.2%
Khan et al. [75]	2020	Common IoT attacks	NSL-KDD	ELM	Accuracy-93.91%
Elsayed et al. [21]	2020	DDoS	CICDDoS2019	AE with RNN	Accuracy-99%
Yuan et al. [12]	2020	Insider Threat	CERT v4.2	LSTM + CNN	AUC-0.9449
Ahmed et al. [58]	2020	Zero-day attacks	CTU-13	ANN	Accuracy (ANN-99.6%)
Doriguzzi-Corin et al. [23]	2020	DDoS	ISCX2012, CICIDS2017, CICIDS2018, UNB201X	CNN	CSECIC2018: Accuracy-98.88% ISCX2012: Accuracy-99.87% CIC2017: Accuracy-99.67% UNB201X: Accuracy-99.46%
Yang et al. [82]	2020	Malicious Traffic	Network Captures	RNN	Accuracy (RNN-98%)
Ramos et al. [71]	2020	Botnet Attacks	ISOT-HTTP, CSE-CICIDS2018	RF, DT, SVM, NB, KNN	CIC-IDS2018: Accuracy (RF, DT-99.99%) ISOT-HTTP: Accuracy (DT-99.90%)
Sethi et al. [101]	2020	Malicious Traffic	ISOT CID, NSL-KDD	DDQN	ISOT CID: Accuracy-96.87% NSL-KDD: Accuracy-83.40%
Singh et al. [111]	2020	Malicious DoH Traffic (at DNS level)	CIRA-CIC-DoHBrw-2020	GB, NB, RF, KNN, LR	Accuracy (RF, GB-100%)
Mohammad et al. [35]	2020	DDoS	UNSW-NB15, UCI datasets	Improved Rule Induction (IRI)	F Score (IRI-93.90%)
Letteri et al. [70]	2020	Malware Attack	MTA KDD 19	MLP using AE optimization or RRw optimization	Accuracy (MLP with RRw opt.-99.60%)
Rendall et al. [48]	2020	Phishing Attack	Private Dataset	SVM, NB, DT, MLP	Accuracy (MLP, DT-86%)
Kim et al. [41]	2020	DDoS	KDD-99, CICIDS2018	CNN, RNN	Accuracy (CNN-99% or more)
Alrashdi et al. [81]	2019	Common IoT attacks	UNSW-NB15	RF	Accuracy (ML-99.34%)
Chawla et al. [146]	2019	IDS	ADFA	RNN, CNN	Time Taken (CNN-GRU 10× faster than LSTM)
Halimaa et al. [130]	2019	IDS	NSL-KDD	SVM, and NB.	Accuracy (SVM-93.95%)
Ongun et al. [98]	2019	Malicious Traffic	CTU-13	LR, RF, and GB	AUC (RF-99%)
De Lucia et al. [91]	2019	Malicious Traffic	Datasets from Stratosphereips.org	SVM and CNN	F-Score (SVM-0.9997)
Filho et al. [32]	2019	DDoS	CICDoS2017, CICIDS2017, CICIDS2018	RF, LR, AdaBoost, Stochastic Gradient Descent, DT, and Perceptron	Accuracy (RF-96%)
Radivilova et al. [30]	2019	DDoS	SNMP-MIB	RF	Accuracy (RF-0.9)
Zhang et al. [116]	2019	IDS	NSL-KDD	AE	F-Score-76.47% Recall-79.47%
Vijayanand et al. [34]	2019	DDoS	CICIDS2017	SVM, Multi-Layer Deep Networks	Accuracy (MLDN-99.99%)
Hu et al. [14]	2019	Insider Threat	Private Dataset	CNN	FAR-2.94% FRR-2.28%
Ullah et al. [76]	2019	Common IoT attacks	Private Dataset	CNN	Accuracy (CNN-97.46%)
Baek et al. [18]	2019	DDoS	Private Dataset	MLP	Accuracy (MLP-50%)
Shi et al. [26]	2019	DDoS	CICIDS2017	LSTM	Accuracy (LSTM-99%)
Sabeel et al. [20]	2019	DDoS	CICIDS2017	DNN, LSTM	TPR (DNN-99.8%) TPR (LSTM-99.9%)
Wu et al. [117]	2019	IDS	UNSW-NB15, NSL-KDD	CNN, RNN	Binary Class: Accuracy-99.24% Multiclass: Accuracy-99.05%
Tama et al. [148]	2019	IDS	NSL-KDD, UNSW-NB15	rotation forest + bagging	UNSW-NB15: Accuracy-91.27% NSL-KDD: Accuracy-85.8%
Rao et al. [54]	2019	Phishing Attacks	Private Dataset	LSTM + SVM	Accuracy (LSTM + SVM-97.3%)
Min et al. [149]	2018	IDS	ISCX2012	RF, SVM, NN, CNN	Accuracy (RF-99.13%)
Pektas et al. [73]	2018	Botnet Attacks	ISOT HTTP, CTU-13	MLP + LSTM	ISOT: F score-98.8% CTU: F score-99.1%
Ahmad et al. [135]	2018	IDS	NSL-KDD	SVM, RF, ELM	Accuracy (ELM-99.5%)
Shafiq et al. [94]	2018	Malicious Traffic	HIT Trace 1 captures NIMS dataset	BayesNet, NB, AdaBoost, Bagging, PART, C4.5, RF, Random Tree, Sequential Minimal Optimization, oneR, Hoeffding	HIT: Accuracy (PART-97.88%) NIMS: Accuracy (RF-100%)
Park et al. [64]	2018	Malware Traffic	Kyoto 2006+	RF	F-Score (RF-99%)
Chou et al. [83]	2018	Malicious Traffic	NSL-KDD	NNET	Accuracy (NNet-97.65%)
Nguyen et al. [147]	2018	IDS	UNSW-NB15, KDD-99, NSL-KDD	NNET	Accuracy (KDD-99-97.11%)
Al-Qatf et al. [114]	2018	IDS	NSL-KDD	SVM, STL	Binary: (Accuracy-84.96%) Multiclass (Accuracy-80.48%)
Millar et al. [88]	2018	Malicious Traffic	UNSW-NB15	NNET	F-Score (Flow image-94.2%)
Wu et al. [67]	2018	Malware Traffic	EMBER	DQN, SARSA, Double DQN	Accuracy (DQN-93.5%)
Li et al. [49]	2018	Phishing Attacks	50K-PD, 50K-IPD	GBDT + XGBoost + LightGBM	50K-PD: Accuracy-97.3% 50K-IPD: Accuracy-98.6%
Vanhoenshoven et al. [104]	2017	Malicious Traffic	Malicious URLs	KNN, RF, SVM, DT, NB, MLP	Accuracy (RF-97%)
Kumar et al. [141]	2017	IDS	Wireshark Network Captures	ensemble of RF, PART and JRIP	Accuracy-98.2%
Anderson et al. [151]	2017	Malware Traffic	Captured TLS encrypted sessions	Linear Regression, l1/l2-LR, DT, RF ensemble, SVM, MLP	Accuracy (LR-99.92%)
Almseidin et al. [125]	2017	IDS	KDD-99	J.48, RF, Random Tree, Decision Table, NB, Bayes Network, MLP	Accuracy (RF-93.77%)
Ghanem et al. [131]	2017	IDS	Five datasets gathered from an IEEE 802.11 and a private dataset	SVM	DR, OSR (on all datasets-100%)
Xu et al. [90]	2017	Malicious Traffic	Network Capture	RF, LR	Kernet: DR(RF-100%) User-level: DR(RF-99%)
Tama et al. [78]	2017	Common IoT attacks	CIDDS-001, UNSW-NB15, GPRS-WEP, GPRS-WPA2	DNN	CIDDS-001: Accuracy-94.17% UNSW-NB15: Accuracy-99.99% GPRS-WEP: Accuracy-82.89% GPRS-WPA2: Accuracy-94%
Yuan et al. [16]	2017	DDoS	ISCX 2012	RNN	Error Rate (RNN-2.103%)
Amira et al. [136]	2017	IDS	NSL-KDD	NB, DT, NBTree, BFTree, J.48, RFT, MLP	Accuracy (MLP-98.54%)
Niyaz et al. [27]	2017	DDoS	Network Capture	SAE	Accuracy (SAE-95.65%)
Belavagi et al. [124]	2016	IDS	NSL-KDD	LR, SVM, NB, RF	Accuracy-(RF-99%)
Mehmood et al. [132]	2016	IDS	KDD-99	SVM, NB, J.48, Decision Table	Accuracy (J.48-–99%)
Alrawashdeh et al. [122]	2016	IDS	KDD-99	RBM, DBN, DBN + LR	Accuracy (DBN + LR-97.9%)
Robinson et al. [38]	2016	DDoS	CAIDA conficker, CAIDA DoS, KDD-99	NB, RF, MLP, voting, BayesNet, IBK, J.48	Accuracy (RF-100%)
Thabtah et al. [47]	2016	Phishing	Datasets from UCI	NNet	Accuracy-93.06%
Tahir et al. [142]	2015	IDS	NSL-KDD	hybrid of K-means Clustering and SVM	DR-96.26%
Choudhury et al. [126]	2015	IDS	NSL-KDD	BayesNet, LR, IBK, J.48, PART, JRip, Random Tree, RF, REPTree, boosting, bagging, and blending	Accuracy (RF-91.523%)
Niyaz et al. [115]	2015	IDS	NSL-KDD	STL with AE	Accuracy (STL-98%)
David et al. [66]	2015	Malware Attacks	Private Dataset	DBN	Accuracy (DBN-98.6%)
Barati et al. [40]	2015	DDoS	CAIDA USCD 2007	GA + MLP	AUC-0.9991
Abuadlla et al. [121]	2014	IDS	Network Capture	NNET, RBFN	Accuracy-99.4%
Xie et al. [102]	2014	Malicious Traffic	ADFA	SVM	Accuracy (70%), FPR (20% when k = 5)
Mohammad et al. [44]	2014	Phishing Attacks	Private Dataset	ANN	Accuracy (testing set-92.18%)
Beaver et al. [57]	2013	Zero-day Attacks	KDD-99	AdaBoost	Accuracy (AdaBoost-94%)
Devikrishna et al. [120]	2013	IDS	KDD-99	ANN	Successfully detected and classified attacks
Lehnert et al. [144]	2012	IDS	KDD-99	SVM, Clustering, NNET	Error Rate (SVM-2.79%)
Sharma et al. [143]	2012	IDS	KDD-99	K-means clustering via NB	DR-99%
Gogoi et al. [137]	2012	IDS	TUIDS, NSL-KDD, KDD-99	Clustering	TUIDS Packet level: accuracy = 99.42%. KDD: accuracy = 92.39%. NSL-KDD: accuracy = 98.34%
Hasan et al. [118]	2012	IDS	DARPA 1998	NNET	Accuracy (NNet-92%)
Wattanapongsakorn et al. [139]	2011	IDS	Network Capture	DT, Bayesian, Ripple Rule Back Propagation Neural Network	DR (DT-95.5%)
Al-Janabi et al. [123]	2011	IDS	KDD-99	ANN	DR (ANN-91%)
Sun et al. [87]	2010	Malicious Traffic	Network Capture	SVM, RBFNN, PNN	Accuracy (PNN-88.18%)

**Table 3:** ML and DL algorithms evaluated in the reviewed papers.

Algorithm	Papers That Applied It	No. of Articles	Problem Domains	Performance (Highest Accuracy)
SVM	[10,28,34,42,48,51,54,60,71,87,91,99,102,104,124,127,128,130,131,132,135,142,144,149,151]	26	Insider Threat, DDoS, Malware, Botnet, Malicious Traffic, IDS, Phishing	93.95% (IDS)
DT	[32,43,48,51,60,71,99,104,128,132,136,139,151]	13	Insider Threat, DDoS, Phishing, Malware, Botnet, Malicious Traffic, IDS	100% (Malicious Traffic)
RF	[28,30,32,38,43,51,60,64,71,81,90,94,98,99,104,108,111, 124,125,126,127,128,135,136,148,149,151]	27	DDoS, Phishing, Malware, Botnet, IoT Network, Malicious Traffic, DNS Level Attack, IDS	100% (Malicious Traffic, DDoS)
NB	[38,42,48,51,60,71,94,99,104,111,124,125,127,128,130, 132,136,143,145]	19	DDoS, Malware, Botnet, Malicious Traffic, DNS Level Attack, IDS, Phishing	90% (Malicious Traffic)
KNN	[71,99,104,111,127,128]	6	Botnet, Malicious Traffic, DNS Level Attack, IDS	100% (Malicious Traffic)
MLP	[18,34,38,40,48,51,73,104,125,151]	11	DDoS, Malware, Botnet, Malicious Traffic, IDS, Phishing	99.60% (Malware)
ELM	[75,135]	2	IDS	99.5% (IDS)
LR	[32,90,98,111,124,126,128,151]	8	DDoS, Malware, Malicious Traffic, DNS Level Attack, IDS	99.92% (Malware)
J.48	[38,125,126,132,133,136]	6	DDoS, IDS	99.66% (IDS)
ANN	[44,58,120,123,128,133]	6	Phishing, Zero-Day, IDS	99.6% (Zero-Day)
RNN	[10,16,21,24,41,42,82,106,117,146]	10	Insider Threat, DDoS, Malicious Traffic, IDS	100% (Insider Threat)
CNN	[23,24,41,62,74,76,91,117,146,149]	10	Insider Threat, DDoS, Malware, IoT Network, Malicious Traffic, IDS	99% (DDoS)
DNN	[10,20,78,84]	4	Insider Threat, DDoS, IoT Network, Malicious Traffic	99.99% (IoT Network)
LSTM	[20,24,26,54,73,84,110]	7	DDoS, Botnet, IoT Network, Malicious Traffic, Phishing	99% (DDoS)
CNN-LSTM	[12,24]	2	Insider Threat, DDoS	99.48% (DDoS)
AE	[21,115,116]	3	DDoS, IDS	99% (DDoS)

**Table 4:** Network traffic datasets used in the reviewed papers.

Dataset	Articles	Number	Last Time Dataset Used	Publicly Available
DARPA-1998	[118]	1	2012	[119]
KDD-99	[38,41,57,120,122,123,125,132,137,143,144,147]	12	2018	[39]
NSL-KDD	[28,74,75,83,101,114,115,116,117,124,126,130,135,136,137,142,145,147,148]	19	2021	[29]
UNSW-NB15	[35,78,81,88,117,147,148]	7	2020	[36]
CICIDS-2017 or 2018	[20,23,26,32,34,41,60,71]	8	2020	[17]
CTU-13	[58,73,89,98]	4	2021	[59]
IoTID 20	[108,109,110]	3	2021	[107]
Kyoto 2006+	[64]	1	2018	[65]
CERT v6 or v4	[10,12]	2	2021	[11,13]
SNMP-MIB	[30]	1	2019	[31]
ISCX 2012 or 2016	[16,23,96,149]	4	2020	[17,97]
ADFA	[102,146]	2	2019	[103]
CAIDA	[38,40]	2	2016	[37]
ISOT CID	[99,101]	2	2021	[100]
ISOT HTTP	[71,73]	2	2020	[72]
Malicious URLs Dataset	[104]	1	2021	[105]
EMBER	[67]	1	2018	[68]
CICDDoS2019 or CICDoS2017	[21,32,42]	3	2020	[22,33]
USTCTFC2016	[62]	1	2016	[63]
GPRS WPA2/WEP	[78]	1	2017	[80]
MTA KDD 19	[70]	1	2020	[69]
LITNET-2020	[84]	1	2020	[85]
CIRA-CIC-DoHBrw-2020	[111]	1	2020	[112]
Bot-IoT	[128]	1	2019	[129]
Kaggle Datasets	[43,51]	2	2021	[52,154]
UCI Datasets	[35,47,145]	3	2021	[155]
